# When minutes matter: A university emergency notification system dataset

**DOI:** 10.1016/j.dib.2021.106910

**Published:** 2021-02-26

**Authors:** Mindy Menn, Caroline Payne-Purvis, Beth H. Chaney, J. Don Chaney

**Affiliations:** a Texas Woman's University, United States; b Auburn University, United States; c The University of Alabama, United States

**Keywords:** Health education, Health promotion, Public health, Emergency notification system, College students, Colleges and universities

## Abstract

The data presented in this data article were collected at three points in the 2012–2013 academic year; Fall 2012, Spring 2013, and Summer 2013 from undergraduate students enrolled in an introductory health education course at a large university in the United States. The data regarding undergraduate students’ perceptions of and experiences with the campus emergency notification system were ascertained using a self-administered online-delivered survey instrument. The data included in the Mendeley Data repository affiliated with this data article encompass closed- and open-ended responses from 746 undergraduate students. Closed-ended questions included items based on central constructs from Technology Acceptance Model research–perceived usefulness, perceived ease of use, attitudes toward use, and behavioral intention. Survey questions also assessed students’ actual use of emergency notification messages, students’ perceived self-efficacy to respond to future potential emergency notifications, and demographics and technology use characteristics. This research team asked open-ended questions to collect students’ ideas for systematic improvement in their own words. Descriptive statistics for demographic variables, participant characteristic variables, and scale variables were conducted in SPSS 27 and are provided in tables. The open-ended question response frequencies were also calculated in SPSS 27 and are provided within a supplemental PDF.

To date, no data pertaining to an institution of higher education's emergency notification system are published for open access use. This article provides open access data and surveys that campus emergency planners, researchers, and health education specialists can use to inform emergency communication plans, improve the content of critical campus alert messages, structure future emergency notification studies, and frame future emergency notification system evaluations. This research team anticipates these data will help campus emergency personnel craft more effective messages and optimize their channel mixture to make emergency notifications reach and resonate with students in situations when minutes matter. The data for this article are hosted in a .csv file for widespread access in the following Mendeley Data repository: https://data.mendeley.com/datasets/6jdwfbwzk5/1

**Specifications Table**

**Table:** 

Subject	Public Health and Health Policy
Specific subject area	Health Education/Health Promotion
Type of data	Tables
How data were acquired	Data were acquired through an online-delivered, self-administered survey instrument through Qualtrics survey software. The survey was conducted in Fall 2012, Spring 2013, and Summer 2013. The link to the survey was provided to students enrolled in the selected introductory health education course. The survey link was not publicly distributed or advertised. Recruited participants accessed the survey via the link in their course management system and completed the survey on the device and in the location of their choice. The surveys were not timed, responses were anonymous, and survey protection settings were enacted so that participants could only take the survey once. Two PDFs include the primary survey instruments; one from Fall 2012, and one from Spring and Summer 2013 and are provided as supplemental material files. Contact information was redacted from the supplemental files.
Data format	Raw and Analyzed
Parameters for data collection	These data were collected from undergraduate students (freshmen, sophomores, juniors, and seniors) enrolled in an online health education/health promotion course at one large university in the southeastern United States. The course was open to students across all majors as one option to fulfill an elective requirement.
Description of data collection	The data in this article were collected from undergraduate students enrolled in a foundational health education course at a large university through an online survey. The raw data were collected at three points, compiled, and provided in one .csv file.
Data source location	Institution: University of Florida City/Town/Region: Gainesville, Florida Country: United States of America
Data accessibility	Repository name: Mendeley Data Data identification number: 10.17632/6jdwfbwzk5.1 Direct URL to data: https://data.mendeley.com/datasets/6jdwfbwzk5/1

## Value of the Data

• These data are important to improving campus notification systems, selecting optimal communication channels to reach undergraduate students, and crafting actionable, informative messages to resonate with undergraduate students in emergency situations.
• College and university emergency management directors, health education specialists, emergency communication professionals, and ultimately, future undergraduate students receiving emergency alert messages can benefit from these data.
• Health education specialists and public health practitioners might use these quantitative and qualitative data to inform how to best craft and deliver emergency notifications to undergraduate students. Researchers interested in examining the Technology Acceptance Model in relationship to emergency notification systems may use these pilot data to develop future instruments and frame future experiments.
• Responses to four open-ended items pertaining to the best methods and any additional channels university officials could use to provide emergency information to students could either reinforce current practices or provide ideas for additional communication methods.
• Responses to one item asking students how the university could improve the campus notification system provides insights into preferences and perceptions of a campus emergency notification system in students’ own words.
• On an overarching societal level, these data could save lives. In a life-threatening emergency such as an active shooter on campus, severe weather event, or structure fire, students need rapid and clear communication to inform life or death decisions.
• The potential emergencies examined in 2012 and 2013 (e.g., inclement weather, campus evacuation, gunman on campus, utility failure, shelter-in-place) remain threats to student safety. These situations still threaten students’ safety and security both on the campus where data collection occurred and at institutions of higher education across the United States.
• Data were collected in 2012 and 2013 and are still relevant as all nine notification channels examined initially (e.g., text messages, emails, the university website) are still in use and at the forefront of the emergency notification system.

## 1 Data Description

The data includes three repeated cross sections of undergraduate students. This data article includes one .csv dataset and four supplemental files. Emergency notification systems are mandated across institutions of higher education across the United States yet remain relatively under-evaluated compared to other higher education topics [1], [2], [3], [4], [5], [6]. These data uniquely contribute to the literature by modifying and measuring constructs originating from the technology acceptance model (TAM) [7] as related to emergency notification system use. The seminal Theory of Reasoned Action served as the theoretical base for the TAM [8,9]. As such, the TAM indicates that two primary factors, perceived ease of use and perceived usefulness, are determinants for behavioral intention and behavioral intention is predictive of a person's actual use (and acceptance) of a specific technology.

Across the three data collection points, 785 people responded to the survey invitation. After combining the three datasets into one file, researchers removed cases from participants who did not consent to participate (*n* = 1) or who already participated (*n* = 1). The researchers also removed cases from participants who did not identify as undergraduate students at the time of survey participation. Specifically, the team removed 12 cases from participants who self-identified as graduate students (*n* = 6), non-degree seeking students (*n* = 5), or a professional student (*n* = 1). This research team also removed cases from 24 participants who did not identify their academic classification.

After reviewing all responses, one additional case was removed for including off-topic vulgarity in response to all of the open-ended questions. The final dataset .csv file includes data from 746 undergraduate students.

In Fall 2012, Spring 2013, and Summer 2013 students enrolled in an undergraduate health education/promotion course were asked to complete an online-delivered survey instrument through Qualtrics. The instrument disseminated in Fall 2012 included one matrix question measuring perceived ease of use, one matrix question measuring perceived usefulness of the campus emergency notification system, one matrix question measuring five different aspects of behavioral intentions, three questions measuring participants’ attitudes toward the campus emergency notification system, one matrix question measuring self-efficacy in responding to 13 different potential campus emergencies, three open ended questions regarding the best methods the university could use to contact students on and off campus and how the university could improve the system, 16 demographic questions, and four questions measuring students’ actual use of the campus notification system. Due to the multifaceted nature of self-reported hypothetical behavior in campus emergency situations, the research team measured actual use in two ways; 1) Receipt of one or more campus alert messages and 2) Response to campus alert messages. Survey items organized by construct are presented in Table 1. Table 1 also includes the Cronbach's alpha values for the respective constructs as analyzed in the combined sample (*N* = 746).

**Table 1:** Items organized by construct.

Construct	Items
Perceived Ease of Use	To what extent do you agree or disagree with the following statements?
α = 0.87	**easeofuse1:** I think that UF Alerts are easy to use.
	**easeofuse2:** I think that UF Alerts are clear and understandable.
	**easeofuse3:** Learning how to use the UF Alert system is easy to interact with.
	**easeofuse4:** The UF Alert system is easy to interact with.
Perceived Usefulness	To what extent do you agree or disagree with the following statements?
α = 0.82	**usefulness1:** As a student at the University of Florida (UF), I find that UF Alerts are useful.
	**usefulness2:** The UF Alert system enables me to access the most RECENT updates about emergency situations occurring on or near the University of Florida campus.
	**usefulness3:** The UF Alert system enables me to access the most ACCURATE updates about emergency situations occurring on or near the University of Florida campus.
	**usefulness5:** The University of Florida needs an emergency notification to inform students, faculty, and staff about emergency situations occurring on or near the University of Florida campus.
Self-Efficacy	To what extent do you agree or disagree with the following statements? If I received a UF Alert about an on campus _____, I would know how to respond.
α = 0.95	**se1respondshelter:** Shelter-In-Place
	**se2respondevac:** Building Evacuation
	**se3respondcampusevac:** Campus Evacuation
	**se4respondchemhazmat:** Chemical or Hazardous Material Spill
	**se5respondmissing:** Missing Person
	**se6respondsuicide:** Suicide Threat or Attempt
	**se7respondutility:** Utility Failure
	**se8respondbomb:** Bomb Threat
	**se9respondfire:** Fire
	**se10respondgunman:** Gunman on Campus
	**se11respondtrespass:** Trespass Notice
	**se12respondtornado:** Tornado
	**se13respondinclement:** Inclement Weather Closing
Behavioral Intention to Use	To what extent do you agree or disagree with the following statements?
α = 0.59	**intendread1:** I intend to read all future UF Alert messages I receive.
	**intendfollow2:** I intend to ``follow'' UF Alerts on Twitter.
	**intendlike3:** I intend to ``like'' UF Alerts on Facebook.
	**intendunsubscribe4:** I intend to unsubscribe from UF Alert text messages.
	**intendignore5:** I intend to ignore all future UF Alert messages.
Attitude Toward Use	To what extent do you agree or disagree with the following statements? Sending a UF Alert through each of the following channels is a good idea.
α =0.87	**attitudeUFhomepage1:** The University of Florida homepage
	**attitudetext2:** Text messaging
	**attitudeemail3:** Email
	**attitudeclassphones4:** IP telephones and speakers (i.e., phones and speakers installed in classrooms)
	**attitudefacebook5:** Facebook
	**attitudetwitter6:** Twitter
	**attituderssfeed7:** RSS feed
	**attitudeUFrumorcontrol8:** University of Florida Rumor Control Hotline
Actual Use: Receipt	**actualuse1:** Have you ever received and/or read at least one UF alert message since you have been a student at UF?
Actual Use: Response	Did you respond to any of the following UF Alerts? (By respond we mean did you take any actions such as leaving campus, evacuating a building, avoiding an area of campus, contacting a law enforcement agency, or alerting other individuals to the notification).
α =0.98	**userespond1:** UF Alert Ref armed subject Shands Gainesville, Garage10. Officers clearing location. Please refer to http://www.ufl.edu for details.
	**userespond2:** (1 of 2) UF Alert Ref incident at Shands Gnvl in Garage10 armed Black male subject believed to have left the campus area Call 352–392–1111
	**userespond3:** (2 of 2) with info 2 of 3 message
	**userespond4:** (1 of 2) UF Alert Reference armed disturbance reported at Shands Gainesville in Garage 10. Two subjects, one black male armed with handgun.
	**userespond5:** (2 of 2) 1 of 2 messages
	**userespond6:** UF Alert Location of incident is Gar10 Shands Gainesville no injuries reported at this time.
	**userespond7:** UF Alert 2 subjects detained, 1 black male white shirt with hand gun at large. Report info to 352–392–1111
	**userespond8:** UF Alert Reported armed 2 males subj's unk. race at shands Gar.10 unknown intent. UPD on scene. Report info to 352–392–1111
	**userespond9:** UF Alert Reference the Bomb Threat at Shands UF the threat was a hoax and two are now in custody.
	**userespond10:** UF Alert Shands main campus No suspicious devices located Officers are clearing the area Call 352 392–1111 with any information

*Note.* The researchers originally measured perceived usefulness with five items and the Cronbach's alpha was 0.53. Four items were worthy of retention, but one item (usefulness4) was not retained for analysis as removal increased the Cronbach's alpha to 0.82.

In Spring and Summer 2013, the survey instrument included all of the above items and added two additional open-ended response items. These open-ended items were introduced to identify additional methods university personnel could use to optimally communicate emergency information to students on and off campus. One PDF, including SPSS output from the four open-ended questions, is included in this data article's supplemental material. One meta-info item was embedded in both iterations of the survey instrument and disclosed to participants within the informed consent process. The meta-info item automatically collected data regarding participants’ browser, browser version, operating system, and screen resolution. Qualtrics also automatically collected, and the .csv file includes system-generated meta-info for each response's start date, end date, and duration (in seconds). Both survey instruments (Fall 2012 and Spring/Summer 2013) and a codebook are attached as supplemental materials.

Table 2 includes select demographic and participant characteristics for the 746 undergraduate students. The demographic characteristics reported in Table 2 include participants’: Sex, Academic Classification, Current Residence Type, High Speed Internet availability at Current Residence, if students have an Unlimited Text Message/SMS Plan, the Number of Texts Sent Per Day and the Number of Texts Received Per Day.

**Table 2:** Participant characteristics.

	Fall 2012	Spring 2013	Summer 2013	Across Semesters
	*n* *=* *410*	*n* *=* *273*	*n* *=* *63*	*N* = 746
Characteristic	*n* (%)	*n* (%)	*n* (%)	*N* (%)
**Sex**				
Female	293 (71.46)	207 (75.82)	44 (69.84)	544 (72.92)
Male	116 (28.30)	65 (23.81)	18 (28.57)	199 (26.68)
Intersex	1 (0.24)	0 (0.00)	1 (1.59)	2 (0.27)
Missing	0 (0.00)	1 (0.37)	0 (0.00)	1 (0.13)
**Academic Classification**				
Freshman	38 (9.27)	41 (15.02)	15 (23.81)	94 (12.60)
Sophomore	124 (30.24)	90 (32.97)	7 (11.11)	221 (29.62)
Junior	145 (35.37)	63 (23.08)	23 (36.51)	231 (30.97)
Senior	103 (25.12)	79 (28.94)	18 (28.57)	200 (26.81)
**Current Residence Type**				
On campus dormitory	61 (14.88)	60 (21.98)	14 (22.22)	135 (18.10)
Off-campus dormitory	3 (0.73)	13 (4.76)	0 (0.00)	16 (2.14)
Apartment	245 (59.75)	142 (52.01)	33 (52.38)	420 (56.30)
House	89 (21.71)	51 (18.68)	16 (25.40)	156 (20.91)
Other	12 (2.93)	6 (2.20)	0 (0.00)	18 (2.41)
Missing	0 (0.00)	1 (0.37)	0 (0.00)	1 (0.13)
**High-Speed Internet at Current Residence**				
Yes	402 (98.05)	265 (97.07)	61 (96.83)	728 (97.59)
No	3 (0.73)	4 (1.47)	2 (3.17)	9 (1.21)
I'm not sure	5 (1.22)	3 (1.10)	0 (0.00)	8 (1.07)
Missing	0 (0.00)	1 (0.37)	0 (0.00)	1 (0.13)
**Unlimited Text Message/SMS Plan**				
Yes	389 (94.88)	257 (94.14)	60 (95.24)	706 (94.64)
No	14 (3.41)	13 (4.76)	2 (3.17)	29 (3.89)
I don't know	7 (1.71)	2 (0.73)	1 (1.59)	10 (1.34)
Missing	0 (0.00)	1 (0.37)	0 (0.00)	1 (0.13)
**Number of Text Messages Sent Per Day**				
0–10	37 (9.02)	22 (8.06)	6 (9.52)	65 (8.71)
11–20	78 (19.02)	56 (20.51)	10 (15.87)	144 (19.30)
21–50	135 (32.93)	94 (34.43)	16 (25.40)	245 (32.84)
More than 50	143 (34.88)	86 (31.50)	28 (44.44)	257 (34.45)
I don't know	16 (3.90)	14 (5.13)	3 (4.76)	33 (4.42)
Missing	1 (0.24)	1 (0.37)	0 (0.00)	2 (0.27)
**Number of Text Messages Received Per Day**				
0–10	43 (10.49)	27 (9.89)	9 (14.29)	79 (10.59)
11–20	71 (17.32)	51 (18.68)	8 (12.70)	130 (17.43)
21–50	124 (30.24)	92 (33.70)	16 (25.40)	232 (31.10)
More than 50	151 (36.83)	88 (32.23)	27 (42.86)	266 (35.66)
I don't know	19 (4.63)	14 (5.13)	3 (4.76)	36 (4.83)
Missing	2 (0.49)	1 (0.37)	0 (0.00)	3 (0.40)

Table 3 includes frequencies and percentages regarding the devices students reported owning at the time of survey participation. As students could report owning multiple devices, percentages in Table 3 do not add to 100.00%. Since most emergency notifications were sent through electronic communication channels, the researchers emphasized asking students about devices, which could be used to receive messages.

**Table 3:** Device ownership characteristics.

	Fall 2012	Spring 2013	Summer 2013	Across Semesters
	*n* = 410	*n* = 273	*n* = 63	*N* = 746
	*n* (%)	*n* (%)	*n* (%)	*N* (%)
Cell phone with Internet capability (smart phone)	373 (90.98)	253 (92.67)	61 (96.83)	688 (92.10)
Cell phone without Internet capability	86 (20.98)	45 (16.79)	15 (23.81)	147 (19.68)
Desktop Computer	103 (25.12)	77 (28.21)	16 (25.40)	197 (26.37)
Electronic Book Reader	87 (21.22)	79 (28.94)	11 (17.46)	178 (23.83)
Game Console	168 (40.98)	113 (41.39)	25 (39.68)	307 (41.10)
Laptop Computer	393 (95.85)	268 (98.17)	63 (100.00)	725 (97.05)
mp3 Player	331 (80.73)	209 (76.56)	50 (79.37)	591 (79.12)
Netbook Computer	48 (11.71)	30 (10.99)	6 (9.52)	85 (11.38)
Portable Gaming Device	61 (14.88)	42 (15.38)	3 (4.76)	107 (14.32)
Tablet Computer	86 (20.98)	82 (30.04)	12 (19.05)	181 (24.23)

Table 4 includes the Means, Standard Deviations, and sample sizes for responses to the question, “To what extent do you agree or disagree with the following statements? If I received a UF Alert about an on campus ___________________, I would know how to respond.” This matrix question ascertained students’ perceived self-efficacy to respond to 13 different campus safety threats on a 5-point Likert scale ranging from 1 (strongly agree) to 7 (strongly disagree).

**Table 4:** Perceived self-efficacy to respond to campus safety threats.

	Fall 2012			Spring 2013			Summer 2013			Across Semesters		
Threat	*M*	*SD*	*n*	*M*	*SD*	*n*	*M*	*SD*	*n*	*M*	*SD*	*N*
Bomb Threat	3.69	1.84	407	4.06	2.04	269	4.16	2.06	63	3.86	1.94	740
Building Evacuation	2.82	1.69	408	3.01	1.91	272	2.79	1.76	62	2.89	1.78	743
Campus Evacuation	3.06	1.81	405	3.29	1.95	273	3.11	1.94	63	3.14	1.88	742
Chemical or Hazardous Material Spill	3.84	1.80	409	4.18	1.97	272	4.11	2.11	63	3.98	1.90	745
Fire	2.91	1.70	409	3.19	2.01	271	3.00	1.97	62	3.02	1.84	743
Gunman on Campus	3.61	1.87	409	3.89	2.03	272	3.83	2.14	63	3.73	1.96	745
Missing Person	3.67	1.62	408	3.77	1.85	272	4.14	1.83	63	3.74	1.73	744
Shelter-in-Place	3.93	1.77	407	4.12	1.98	273	4.05	1.89	63	4.00	1.86	744
Suicide Threat or Attempt	3.92	1.70	409	4.11	1.87	273	4.56	1.78	63	4.04	1.78	746
Tornado	3.52	1.84	408	3.82	1.96	273	3.54	1.94	63	3.63	1.90	745
Trespass Notice	3.76	1.69	407	3.98	1.87	272	4.16	1.76	63	3.87	1.77	743
Utility Failure	3.94	1.67	408	4.19	1.85	271	4.38	1.86	63	4.06	1.76	743
Weather Closing	3.34	1.79	406	3.59	1.96	273	3.24	1.95	62	3.42	1.87	742

Table 5 includes the means, standard deviations, and sample sizes of students in response to items measuring students’ perceived ease of use, perceived usefulness, and behavioral intentions related to using the UF Alert system. Perceived ease of use, perceived usefulness, and behavioral intentions were measured on a 7-point Likert scale ranging from 1 (strongly agree) to 7 (strongly disagree).

**Table 5:** Means with standard deviations of college students’ perceived ease of use, perceived usefulness, and behavioral intention perceptions toward a campus notification system across three semesters.

	Fall 2012			Spring 2013			Summer 2013			Across Semesters		
	*M*	*SD*	*n*	*M*	*SD*	*n*	*M*	*SD*	*n*	*M*	*SD*	*N*
**Perceived Ease of Use**												
UF Alerts are easy to use	2.06	1.16	410	2.18	1.25	271	2.29	1.39	63	2.13	1.22	744
UF Alerts are clear and understandable	2.37	1.41	409	2.39	1.36	271	2.48	1.35	63	2.38	1.39	743
Learning how to use the UF Alert system was easy for me	2.42	1.41	409	2.62	1.56	271	2.76	1.57	63	2.52	1.48	743
The UF Alert system is easy to interact with.	2.88	1.59	409	3.21	1.70	271	2.95	1.67	63	3.01	1.64	743
**Perceived Usefulness**												
As a student at UF I find that UF Alerts are useful	2.01	1.22	410	2.29	1.42	272	2.06	1.31	63	2.12	1.31	745
The UF Alert system enables me to access the most RECENT updates about emergency situations occurring on or near the UF campus	2.10	1.26	409	2.37	1.39	273	2.21	1.35	63	2.21	1.32	745
The UF Alert system enables me to access the most ACCURATE updates about emergency situations occurring on or near the UF campus	2.17	1.24	409	2.51	1.41	273	2.37	1.24	63	2.31	1.31	745
If I could, I would unsubscribe from the UF Alert email system	4.87	2.04	407	4.89	1.96	273	5.05	1.91	63	4.89	2.00	743
The University of Florida needs an emergency notification system to inform students, faculty, and staff about emergency incidents	2.07	1.37	409	2.23	1.50	272	1.84	1.14	63	2.11	1.41	744
**Behavioral Intention**												
I intend to read all future UF Alert messages I receive	1.84	1.14	409	2.15	1.45	272	2.10	1.33	63	1.97	1.29	744
I intend to “follow” UF Alerts on Twitter	4.62	1.92	408	4.70	1.97	271	5.19	1.88	63	4.70	1.94	742
I intend to “like” UF Alerts on Facebook	4.28	1.96	408	4.45	2.00	273	4.63	2.03	63	4.37	1.98	744
I intend to unsubscribe from UF Alert text messages (Reverse coded)	2.35	1.67	407	2.46	1.72	271	2.17	1.66	63	2.38	1.69	741
I intend to ignore all future UF Alert messages (Reverse coded)	2.11	1.50	408	2.31	1.63	273	1.75	1.14	61	2.15	1.53	742

## 2 Experimental Design, Materials and Methods

The data presented in this data article were collected within the overarching Health Education Research Experience (HERE) program as described in more detail in a separate data article [10]. The population of interest for these data encompassed degree-seeking and enrolled undergraduate students across classifications (freshmen, sophomores, juniors, and seniors). The research team intentionally selected undergraduates as the population of interest, due to the relatively high percentage of undergraduate students who lived and attended classes on campus. This selection was supported by The Carnegie Classification of Institutions of Higher Education who classified the institution's Enrollment Profile as “Majority undergraduate” in the report two years prior to data collection [11].

For research participation credit purposes, all students enrolled in the selected foundational health education course were eligible to participate in Fall 2012. Students were recruited through the Health Education Research Experience activity options provided in the course's learning management system assignments. Data were collected and stored within Qualtrics survey software. In Spring and Summer 2013, students were recruited in the same manner and all students who had not previously participated were eligible to participate. Students who previously participated or who elected to not participate were eligible to complete an alternative assignment for equal course credit.

## Ethics Statement

The University's Institutional Review Board reviewed and approved all related materials (U-1061–2012). The authors confirm that informed consent was obtained from all participants prior to beginning the survey. Participants were not required to answer any question and could quit taking the survey at any time. Participants’ responses are completely anonymous and cannot be connected to the participants. To reduce perceptions of coercion, the primary researcher was not the instructor of record for the course during the data collection period.

## CRediT Author Statement

**Mindy Menn:** Conceptualization, Methodology, Writing-Original draft preparation, Data Curation; **Caroline Payne-Purvis:** Conceptualization, Methodology, Writing - Review & Editing; **Beth H. Chaney:** Supervision, Writing - Review & Editing; **J. Don Chaney:** Supervision, Project administration.

## Declaration of Competing Interest

The authors declare that they have no known competing financial interests or personal relationships, which have, or could be perceived to have, influenced the work reported in this article.

## Appendix Supplementary materials

Image, application 1

Image, application 2

Image, application 3

Image, application 4
