# Supplementary material for: When minutes matter: A university emergency notification system dataset
Source: Data Brief. 2021 Feb 26;35:106910. doi: 10.1016/j.dib.2021.106910 (PMC8010384; doi:10.1016/j.dib.2021.106910)
Supplement: Supplementary file 1 [file mmc1.pdf]

Frequency tables for open-ended questions in *When Minutes Matter: A University Emergency Notification System Dataset by Menn, Payne-Purvis, Chaney, and Chaney*

Table 1

Raw responses to: What additional methods could university officials use to provide emergency information to you while you are OFF campus?

Response	*n*	%
MISSING	555	74.4%
All methods used now are fine.	1	0.1%
An automated phone call	1	0.1%
announce the emergency information on the RTS buses	1	0.1%
automated mass call	1	0.1%
automated phone call	1	0.1%
automated phone call updates, text messages	1	0.1%
Automated phone calls	1	0.1%
Automated telephone calls	1	0.1%
Be informative	1	0.1%
By posting UF alerts on the UF homepage and on ISIS webpage.	1	0.1%
Cell phones	1	0.1%
certain alarm systems throughout gainesville that note crime on campus/ around town	1	0.1%
communicate with communities that are majority students	1	0.1%
Continue sending emails/text messages	1	0.1%
Continue with texts and e-mails	1	0.1%
Don't know	1	0.1%
E-Learning notification	1	0.1%
E-mail	1	0.1%
E-Mail	1	0.1%
email	5	0.7%
Email	1	0.1%
email and ohone are best, news if the situation is serious enough	1	0.1%
Email and text message are very effective ways	1	0.1%

emailing or phone calls	1	0.1%
emails	3	0.4%
emails, texting	1	0.1%
emergency contact numbers	1	0.1%
emergency TV notification system that overrides normal TV broadcast	1	0.1%
facebook	2	0.3%
Facebook	1	0.1%
facebook instant messaging	1	0.1%
Facebook, bus drivers	1	0.1%
Facebook, Text Message	1	0.1%
Give better details in the text messages.	1	0.1%
have an app that has detailed updates, rather than a brief text message that isn't always helpful	1	0.1%
have classes or open speeches about what to do in emergencies	1	0.1%
I don't know	1	0.1%
i dont know	1	0.1%
I just like the text and email	1	0.1%
I think it's efficient	1	0.1%
I think that the current methods are usefu;.	1	0.1%
I think that the officials are doing a pretty good job at providing emergency information	1	0.1%
I think the texts work	1	0.1%
i think they covered all the ways	1	0.1%
i'm not sure!	1	0.1%
Im not too sure	1	0.1%
Information via rts sevices	1	0.1%
ISIS or MyUFL alerts	1	0.1%
Just keep the text messages and emails	1	0.1%
just more detailed mesages.	1	0.1%
just UF alerts are fine with me	1	0.1%
Local News stations.	1	0.1%
Mainly email and text	1	0.1%
maybe a phone call depending on the severity of the situation	1	0.1%

maybe alert nearby student housing offices (apartments, Trimark, etc)	1	0.1%
messages on the buses	1	0.1%
messenging, emails.	1	0.1%
More clear text messages	1	0.1%
More detailed alerts.	1	0.1%
More detailed emails.	1	0.1%
more texts	1	0.1%
n/a	5	0.7%
N/A	6	0.8%
n/a - believe text is still best	1	0.1%
na	1	0.1%
NA	1	0.1%
news	2	0.3%
Newspapers	1	0.1%
No additional methods	1	0.1%
No Answer	1	0.1%
no comment	1	0.1%
no other methods	1	0.1%
none	13	1.7%
None	6	0.8%
NONE	1	0.1%
none besides the texts.	1	0.1%
None I can think of.	1	0.1%
None, I think text messages and emails are already very efficient.	1	0.1%
None, UF alerts are ok.	1	0.1%
none.	1	0.1%
Not sure	1	0.1%
nothing	3	0.4%
nothing more	1	0.1%
Notify local radio stations to broadcast the alert.	1	0.1%
Notify sorority houses personally for messages pertaining to events nearby	1	0.1%
PA speakers	1	0.1%
patroling	1	0.1%
phone	2	0.3%
Phone	1	0.1%

Phone call with recorded emergency message	1	0.1%
phone calls	1	0.1%
Phone calls	1	0.1%
Phone Calls	1	0.1%
Possible recorded calls reporting the incidence	1	0.1%
Providing some sort of video or a more detailed message	1	0.1%
Public Service announcement on gainesville tv.	1	0.1%
Push notifications from UF app	1	0.1%
put info on facebook	1	0.1%
Radio	1	0.1%
Radio announcement on popular stations	1	0.1%
radio stations?	1	0.1%
sakai announcements	1	0.1%
send texts	1	0.1%
Sending automated voice messages.	1	0.1%
Sending emails	1	0.1%
signs	1	0.1%
sirens for emergencies	1	0.1%
Social Media	1	0.1%
Social networks	1	0.1%
television	2	0.3%
Television	2	0.3%
Tell us to remain off campus if need be.	1	0.1%
text	2	0.3%
Text	1	0.1%
text is sufficient	1	0.1%
Text message are fine, as long as they have a clear purpose and what needs to be done as an actual response``	1	0.1%
text messages	3	0.4%
Text messages	1	0.1%
Text messages and emails	2	0.3%
text messages are the best method	1	0.1%
text messages are the best way	1	0.1%
Text messages.	1	0.1%
Text messages/e-mails are sufficient	1	0.1%

Text messaging and social networking have that covered. As well as e-mail alerts.	1	0.1%
Text messaging is a really affective method	1	0.1%
Text messaging.	1	0.1%
text/email	1	0.1%
Texting works just fine.	1	0.1%
Texting/Email	1	0.1%
Texts/Social Media Sites	1	0.1%
The ones in place are perfect as well.	1	0.1%
the text messages and email is fine	1	0.1%
these seem fine	1	0.1%
They could leave voicemails	1	0.1%
tv	1	0.1%
TV	2	0.3%
tv broadcasts	1	0.1%
tv, radio	1	0.1%
TV, radio	1	0.1%
TV's	1	0.1%
Uf Channel, radio, etc.	1	0.1%
Use major radio stations.	1	0.1%
Use more texts/emails	1	0.1%
Using the radio.	1	0.1%
Warnings about whether it is safe or not to return to campus within 24 hours	1	0.1%
While off-campus, students can receive a phone call with a brief message that can be machine recorded to alert students.	1	0.1%
Yelling	1	0.1%

Table 2

Raw responses to: What is the best method university officials could use to provide emergency information to you while you are ON campus?

Response	*n*	%
MISSING	123	16.5%
alarm system	1	0.1%
alert	1	0.1%
Although I have never heard them, I suppose the speaker announcement is nice, also the text messages are most beneficial for me .	1	0.1%
announce over speakers or have an alarm system	1	0.1%
announcements	1	0.1%
broadcast	1	0.1%
broadcast on speakers	1	0.1%
broadcasting through all speakers and phones on campus	1	0.1%
By text or email message	1	0.1%
cell phone	3	0.4%
Cell phone	2	0.3%
cell phone (text message)	1	0.1%
cell phones and speakers	1	0.1%
Classroom loudspeaker	1	0.1%
Classrooms	1	0.1%
Communcation though social media	1	0.1%
Digital Billboards	1	0.1%
digital signs	1	0.1%
email	14	1.9%
Email	1	0.1%
Email and Text	1	0.1%
email/text messaging	1	0.1%
emailing	1	0.1%
emails	1	0.1%
emails and text messages	1	0.1%
emails, texts, posters, outdoor speakers	1	0.1%
facebook, text	1	0.1%
Facebook/text messaging	1	0.1%
Giant Megaphone	1	0.1%

have an app that has detailed updates, rather than a brief text message that isn't always helpful	1	0.1%
Have instructors tell you	1	0.1%
Have professors relay information	1	0.1%
have teachers get an email and tell all students	1	0.1%
I am never on campus, but I would still have say through text message. It is a large campus, but majority of students/faculty always have a cell phone on their person.	1	0.1%
I feel that the automotive texting method is the best method.	1	0.1%
i have no idea	1	0.1%
I have not had the chance to visit UF.	1	0.1%
I just like the text and email	1	0.1%
I think it's efficient	1	0.1%
I think text message and email are best.	1	0.1%
I think the text message method is the most efficient.	1	0.1%
igns	1	0.1%
Information while in the class room, bathrooms, and central location loud speakers	1	0.1%
intercom	1	0.1%
Intercom	1	0.1%
Intercoms will cause a greater awareness.	1	0.1%
Intercoms.	1	0.1%
internet	1	0.1%
loud speaker as well as text messages, emails, and police on campus patrolling and letting you know.	1	0.1%
Loud speaker/PA system	1	0.1%
loud speakers	1	0.1%
Loud speakers	2	0.3%
loud speakers and alerters on bus speakers	1	0.1%
loud speakers in classes and buildings	1	0.1%
loudspeaker	1	0.1%
loudspeaker, telephone announcement	1	0.1%

make sure all professors announce the alert to the students in class and send off speak messages through turlington etc.	1	0.1%
Making loudspeaker announcements.	1	0.1%
massive email and text message	1	0.1%
messenging	1	0.1%
More cops on patrol and UF alerts immediately after the acknowledgment of a threat to students	1	0.1%
More direct information	1	0.1%
more speakers	1	0.1%
n/a	2	0.3%
N/A	4	0.5%
no idea	1	0.1%
none	3	0.4%
None	2	0.3%
None I can think of.	1	0.1%
None UF alerts are fine.	1	0.1%
Not Sure	1	0.1%
ON CAMPUS ANNOUNCEMENT	1	0.1%
Outdoor PA system or texts	1	0.1%
outdoor speakers	1	0.1%
Outdoor speakers and Digital billboards	1	0.1%
Over a intercom	1	0.1%
Over speakers	1	0.1%
overhead speakers	1	0.1%
PA system	4	0.5%
PA System	1	0.1%
phone	1	0.1%
Phone	1	0.1%
Phone alters	1	0.1%
Phone Calls	1	0.1%
Providing timely information	1	0.1%
Relay messages on the letter signs of rts buses.	1	0.1%
Safety	1	0.1%
same as above and along with dorm room notifications	1	0.1%
see above	1	0.1%

Send text messages	1	0.1%
send texts	1	0.1%
Sending information via text message.	1	0.1%
Setting off all of the blue lights	1	0.1%
Social Media	1	0.1%
Sound signal	1	0.1%
speaker	1	0.1%
speaker alerts	1	0.1%
Speaker announcement	1	0.1%
speaker system	2	0.3%
Speaker that is in classrooms, because when i am in class i don't look at my phone.	1	0.1%
speakers	4	0.5%
Speakers	3	0.4%
speakers and text messages	2	0.3%
speakers around campus	1	0.1%
speakers in classrooms and text messages	1	0.1%
speakers on campus	1	0.1%
Speakers or text	2	0.3%
speakers or texts	1	0.1%
Speakers, emails, text messages	1	0.1%
speakers, high priority alerts sent to professors and T.A.s, and continue with text message alerts to students	1	0.1%
Speakers, text messages	1	0.1%
Teachers reporting situations in classes and possibly canceling that class if needed	1	0.1%
text	58	7.8%
Text	31	4.2%
TEXT	2	0.3%
text alert	1	0.1%
Text alerts as well as intercom announcements	1	0.1%
text and speakers	1	0.1%
Text and televisions	1	0.1%
text and/or over a intercom	1	0.1%
text message	90	12.1%
Text message	62	8.3%
Text Message	25	3.4%

Text Message alert	1	0.1%
Text Message Alert	1	0.1%
text message alerts	1	0.1%
Text message and email	1	0.1%
TEXT MESSAGE AND SPEAKERS	1	0.1%
Text message asap that way we know what we need to do if it is something bad that has happened	1	0.1%
Text message or alarms	1	0.1%
Text message or announcement, depending on situation	1	0.1%
Text message or call	1	0.1%
Text message or email	1	0.1%
Text Message or Email	1	0.1%
text message or in class notifications	1	0.1%
Text message or Intercom announcements	1	0.1%
text message or pa	1	0.1%
Text Message or PA System	1	0.1%
Text Message or speakers in every classroom	1	0.1%
Text Message with instructions	1	0.1%
text message, email, and loudspeaker announcement	1	0.1%
text message, loud speakers, personally being all around campus alerting students	1	0.1%
Text message.	1	0.1%
Text Message/ Maybe announcements through loudspeakers if possible	1	0.1%
text messages	27	3.6%
Text messages	20	2.7%
Text Messages	7	0.9%
Text messages and loud speakers around campus	1	0.1%
Text messages and speakers in classrooms	1	0.1%
text messages is the best option	1	0.1%
text messages or intercom systems.	1	0.1%
Text messages or loud speaker	1	0.1%
text messages, and maybe intercom	1	0.1%

Text messages, and the announcements on the speakers	1	0.1%
text messages, speakers phone with the announcement.	1	0.1%
text messages, twitter, facebook updates.	1	0.1%
Text messages.	2	0.3%
text messaging	15	2.0%
Text messaging	13	1.7%
Text Messaging	3	0.4%
Text messaging and email	1	0.1%
Text messaging and loud speakers	1	0.1%
Text messaging is best	1	0.1%
Text messaging is effective.	1	0.1%
Text messaging or loud speakers	1	0.1%
Text messaging seems to work most efficiently.	1	0.1%
text messaging, email, twitter	1	0.1%
text messaging, flyers, spreading the word	1	0.1%
Text messaging.	2	0.3%
Text messege	1	0.1%
text or e-mail	1	0.1%
Text or email	1	0.1%
text or loudspeaker	1	0.1%
text or speaker system	1	0.1%
Text or through teachers in classes	1	0.1%
Text updates	1	0.1%
text, email, call from UF alert to my cell phone from a special number set up to denote emergencies "emergency hotline"	1	0.1%
Text, email, or an announcement through Sakai or Isis	1	0.1%
text, speakers	1	0.1%
text.	2	0.3%
Text.	1	0.1%
text/ emails	1	0.1%
text/email	1	0.1%
text/email/campus-wide PA system	1	0.1%
texting	16	2.1%
Texting	8	1.1%

texting alert	1	0.1%
Texting is the quickest way to reach the most number of students	1	0.1%
texting or announcement through speakers	1	0.1%
texts	8	1.1%
Texts	1	0.1%
Texts or anouncements	1	0.1%
Texts sent in a very timely manner	1	0.1%
Texts.	1	0.1%
The best way is probably through text.	1	0.1%
The ones in place are great.	1	0.1%
Through classroom speakers or text message	1	0.1%
through classroom speakers/phones	1	0.1%
Through email	1	0.1%
through professors or text	1	0.1%
Through text	1	0.1%
Through text mesaages	1	0.1%
through text messages and emails because students are always on their phones.	1	0.1%
To send a text message and a speaker announcement that informs the most important information and how one will be safe	1	0.1%
Twitter	1	0.1%
txt messaging	1	0.1%
UF alert	1	0.1%
UF Alert	1	0.1%
UF alerts	1	0.1%
UF alerts through text messaging	1	0.1%
UF campus speakers	1	0.1%
UF Text Message Alerts	1	0.1%
Use more texts/emails	1	0.1%
verbal announcements	1	0.1%
via text messeage	1	0.1%

Table 3

Raw responses to: What is the best method university officials could use to provide emergency information to you while you are OFF campus?

Response	*n*	%
MISSING	83	11.1%
A text message sent to everyone that informs people off campus to not return or return when emergency is handled	1	0.1%
alert	1	0.1%
automated phone call recording	1	0.1%
Automated voicemails	1	0.1%
By text messaging and emails	1	0.1%
calling, text message, facebook, saki, and e-mail.	1	0.1%
cell phone	5	0.7%
Cell phone	1	0.1%
Cell Phone	1	0.1%
cell phone (text message)	1	0.1%
Cell phone as usual	1	0.1%
cell phones	1	0.1%
Cell phones	1	0.1%
Cellphones	1	0.1%
continue text message alerts, webpage alerts, etc.	1	0.1%
Doesn't apply I attend UF online coursed only	1	0.1%
dont know	1	0.1%
e mail/ text	1	0.1%
e-mail	1	0.1%
E-mail	2	0.3%
e-mail or text messaging	1	0.1%
e-mails, text messaging	1	0.1%
email	26	3.5%
Email	8	1.1%
email and text message	1	0.1%
email and text messages	1	0.1%
email or text	1	0.1%
Email or text	1	0.1%
email or text message	1	0.1%
Email or text message	1	0.1%

email or text messaging	1	0.1%
email, text message	1	0.1%
email, texts	1	0.1%
email, webpage	1	0.1%
email/text	1	0.1%
email/text messaging	1	0.1%
emailing and using the uf website	1	0.1%
emails	1	0.1%
Emails	1	0.1%
Emails and Text Messages	1	0.1%
Emails and texts	1	0.1%
emails are good enough	1	0.1%
Even Bigger Giant Megaphone	1	0.1%
facebook	2	0.3%
Facebook	2	0.3%
Facebook notification	1	0.1%
have an app that has detailed updates, rather than a brief text message that isn't always helpful	1	0.1%
I believe the text messaging is the most accurate.	1	0.1%
I don't know	1	0.1%
I feel texting is also the best method off campus for providing emergency information	1	0.1%
I just like the text and email	1	0.1%
I think text message and email are best.	1	0.1%
I think the texting is very effective since most people always carry their cell phones with them.	1	0.1%
I'm a distance-ed student and live in a different city. They should check that before sending alert messages to everyone.	1	0.1%
internet	1	0.1%
more descriptive texts	1	0.1%
N/a	1	0.1%
N/A	1	0.1%
none	3	0.4%
NONE UF ALERTS ARE FINE.	1	0.1%
none. just be transparent	1	0.1%

Not Sure	1	0.1%
Patrol	1	0.1%
patrolling	1	0.1%
phone	2	0.3%
phone alters	1	0.1%
Phone call or text messaging.	1	0.1%
phone call, text message	1	0.1%
Phone call/email/text message	1	0.1%
Phone Calls	1	0.1%
Phone Text	1	0.1%
phone, email	1	0.1%
Phone/text	1	0.1%
radio stations	1	0.1%
see last comment	1	0.1%
send text messages or email	1	0.1%
send texts	1	0.1%
Sending text messages about the situation	1	0.1%
social media	1	0.1%
Social Media	2	0.3%
still text message	1	0.1%
Television	1	0.1%
text	83	11.1%
Text	33	4.4%
TEXT	2	0.3%
text alert or email	1	0.1%
text alerts	2	0.3%
text and email	1	0.1%
text emails	1	0.1%
text massaging	1	0.1%
text mesaging	1	0.1%
text message	119	16.0%
Text message	44	5.9%
Text Message	27	3.6%
TEXT MESSAGE	1	0.1%
Text message / E-mail	1	0.1%
Text Message / Email	1	0.1%
Text Message Alert	2	0.3%
text message alerts	1	0.1%

Text message alerts	1	0.1%
text message and email	4	0.5%
Text message is the best	1	0.1%
text message or email	6	0.8%
text message, email	1	0.1%
Text Message, Email, University of Florida website	1	0.1%
text message, facebook status update	1	0.1%
text message, gainesville radio	1	0.1%
text message/emails	1	0.1%
text messages	32	4.3%
Text messages	17	2.3%
Text Messages	8	1.1%
text messages and email	2	0.3%
text messages and emails	2	0.3%
Text messages and emails	1	0.1%
text messages or automated phone calls	1	0.1%
text messages that are clear	1	0.1%
Text messages, email	1	0.1%
Text messages, Email	1	0.1%
text messages, email, facebook, and twitter	1	0.1%
text messages, emails	1	0.1%
Text messages, emails, RSS, and social networks	1	0.1%
Text messages, social networking sites, and UF affiliated sites (Sakai, webmail, homepage)	1	0.1%
text messages/ e-mail	1	0.1%
text messaging	16	2.1%
Text messaging	8	1.1%
Text Messaging	9	1.2%
text messaging and emails	1	0.1%
text messaging is a great way	1	0.1%
text messaging, email	1	0.1%
text messaging, email, twitter	1	0.1%
Text messaging.	1	0.1%
Text messaging/E-mails	1	0.1%
text messege	1	0.1%
Text msg	1	0.1%
Text or call	1	0.1%

text or email	2	0.3%
Text or email	2	0.3%
text updates	1	0.1%
text, email	3	0.4%
Text, email	1	0.1%
text.	1	0.1%
Text.	1	0.1%
text/email	2	0.3%
texting	23	3.1%
Texting	8	1.1%
Texting/email	1	0.1%
texts	14	1.9%
Texts	1	0.1%
Texts or Facebook	1	0.1%
texts or phone calls!	1	0.1%
Texts/Social Media	1	0.1%
texxting	1	0.1%
Through email	1	0.1%
Through email or text.	1	0.1%
Through Telephone	1	0.1%
Through text message and email	1	0.1%
Through text messages and social media sites because students are always on social networks and their phones.	1	0.1%
Through text, email and facebook	1	0.1%
trext messages	1	0.1%
tv radio	1	0.1%
Twitter	1	0.1%
txt	2	0.3%
UF alert	1	0.1%
UF Alert	1	0.1%
UF alerts	2	0.3%
UF alerts through text messaging	1	0.1%
UF Text Message Alerts	1	0.1%
use facebook	1	0.1%
Use more texts/emails	1	0.1%
via phone	1	0.1%
via text message	1	0.1%

via text messeage	1	0.1%
web page alerts, text messages	1	0.1%

Table 4

Raw responses to: What additional methods could university officials use to provide emergency information to you while you are ON campus?

Response	*n*	%
MISSING	544	72.9%
A plane flying overhead with a banner. Blimps. Balloons. Colored flare guns. Speakers if necessary.	1	0.1%
A certain sound of alarms, or possible physical symbols like flags or personnel	1	0.1%
a smartphone app	1	0.1%
Actually seeing the officers on campus informing students when something occurs	1	0.1%
Alert banners across campus showing real current time issues	1	0.1%
Alerts could be posted on the TVs in the Reitz	1	0.1%
Alerts made by professors	1	0.1%
Alligator	1	0.1%
Announce over speakers	1	0.1%
announce the emergency information on the RTS buses	1	0.1%
announcements in libraries	1	0.1%
app for I phone	1	0.1%
automated phone call updates	1	0.1%
be on campus to tell me.	1	0.1%
Broad-based class announcement and continue the text messaging and e-mail alerts as well	1	0.1%
Call professors	1	0.1%
Campus police officers directing people	1	0.1%
campus siren for worst cases.	1	0.1%
changing signs	1	0.1%
Class announcements	1	0.1%
Class room speakers	1	0.1%
Classroom speakers	1	0.1%
Clearer text messages. Sometimes they can be vague and confusing.	1	0.1%
code system: like blue would mean terrorist attack	1	0.1%

Code words that preview staffers can teach to in coming students	1	0.1%
Computers can read message	1	0.1%
Digital Billboards	1	0.1%
digital boards around campus	1	0.1%
digital signs	1	0.1%
Discussing them on Snap	1	0.1%
Efficiently email	1	0.1%
electric signs	1	0.1%
Email	1	0.1%
emails	1	0.1%
Emergency alert system throughout the entire school	1	0.1%
emergency signs/posts similar to the blue light poles	1	0.1%
Employ the use of speakers in classrooms.	1	0.1%
facebook	1	0.1%
Facebook, online updating system	1	0.1%
flyers	2	0.3%
flyers, poster about what to do in the instances of ememrgencies listed in this survey	1	0.1%
Have electronic billboard signs on campus to display important information. Example: Highway electronic signs.	1	0.1%
Have officers walking around campus.	1	0.1%
Have professors inform us	1	0.1%
Have teachers announce it during class.	1	0.1%
I can't think of any.	1	0.1%
I don't know.	1	0.1%
I dont know	1	0.1%
I dont know.	1	0.1%
I just like the text and email	1	0.1%
I think it's efficient	1	0.1%
I think that the current methods are useful.	1	0.1%
I think UF is doing a pretty good job at providing emergency information.	1	0.1%
I was completely unaware of an intercom system. I think it should be used more often.	1	0.1%

I'm not sure but just keep the text messages	1	0.1%
If necessary, Information over a loud speaker.	1	0.1%
If need to evacuate add places that may be the safest.	1	0.1%
If something happens they should state the precautions us as students should take on trying to avoid the area	1	0.1%
Im not sure	1	0.1%
In classroom speakers	1	0.1%
In person notification(Visiting classrooms)	1	0.1%
Incorporate something in the dorms and dining halls	1	0.1%
info boards.	1	0.1%
Inform the teachers in case of lockdowns in a rapid fashion.	1	0.1%
Information on what to do in a situation because many people are unaware on how to handle a situation safely	1	0.1%
information sessions and procedures for emergencies	1	0.1%
Information through the speakers in the class room, but only if actually an emergency or really important	1	0.1%
internal speaker system	1	0.1%
loud speaker usage for extreme emergencies only	1	0.1%
loud speakers	1	0.1%
Loudspeaker announcements	1	0.1%
Loudspeakers	1	0.1%
make them more detailed messages and have systems for people to be safe.	1	0.1%
Making loudspeaker announcements.	1	0.1%
Maybe the blue emergency posts can light up, or have a screen with information on them	1	0.1%
messenging would be best	1	0.1%
More cops patrolling throughout campus when there is a UF alert and not just on the road ways but also through the rietz lawn, plaza and places where cars cannot drive. More bike cops.	1	0.1%

More information on courses of action to take	1	0.1%
more texts	1	0.1%
n/a	5	0.7%
N/A	5	0.7%
na	1	0.1%
News Channel on tv	1	0.1%
no comment	1	0.1%
No idea	1	0.1%
No other method.	1	0.1%
no other methods	1	0.1%
none	10	1.3%
None	6	0.8%
None I can think of.	1	0.1%
none that i could think of	1	0.1%
none.	1	0.1%
Not sure	1	0.1%
nothing	1	0.1%
nothing i can think of	1	0.1%
nothing more	1	0.1%
officials informing students of risky areas and or people	1	0.1%
Outdoor speakers	1	0.1%
outside speakers or speakers inside main buildings (Reitz Union, Hub, etc.)	1	0.1%
overhead speakers	1	0.1%
PA system	2	0.3%
phone	1	0.1%
Phone Calls	1	0.1%
police	1	0.1%
Police offers in every area on campus to make us aware	1	0.1%
police officers	2	0.3%
Push notifications from UF app	1	0.1%
Put messages on TV or computer screens throughout campus	1	0.1%
Put notices on the screens on campus, news-report style	1	0.1%
Put up signs in large populated areas	1	0.1%

Radio announcement on popular stations	1	0.1%
radio, teachers	1	0.1%
Reaching out to the greek communities to spread the word.	1	0.1%
safe evacuation info	1	0.1%
Sakai/E learning home page	1	0.1%
screens	1	0.1%
Scrolling Marquees/Alarms	1	0.1%
send texts	1	0.1%
signs	1	0.1%
Social media	1	0.1%
some kind of scrolling marquee	1	0.1%
speaker announcements	1	0.1%
speaker/intercom is probably most effective	1	0.1%
speakers	4	0.5%
Speakers	4	0.5%
speakers around campus	1	0.1%
speakers in classrooms	1	0.1%
Speakers in classrooms, not outside of them	1	0.1%
speakers in classrooms?	1	0.1%
Speakers outside and in classrooms	1	0.1%
speakers throughout campus	1	0.1%
Speakers?	1	0.1%
speaking	1	0.1%
teachers	1	0.1%
teachers notify students	1	0.1%
Tell us what they would like us to do about the situation in said message.	1	0.1%
Text message	1	0.1%
Text messages	1	0.1%
Text messages are efficient	1	0.1%
text messages would be the best method	1	0.1%
Text messages/e-mails are sufficient	1	0.1%
Text messaging is best	1	0.1%
Text messaging.	1	0.1%
Texting	1	0.1%

Texting is efficient but I feel they should send a text saying whether or not the suspect has been apprehended.	1	0.1%
texting is the main one	1	0.1%
The ones in place now are perfect.	1	0.1%
The signs that construction workers use on the sides of roads during construction.	1	0.1%
the speakers seem like a good idea	1	0.1%
The text messages and speaker is fine	1	0.1%
these seem fine	1	0.1%
They could have cops outside making people aware of serious situations	1	0.1%
They couldn't.	1	0.1%
through emailing not texting because it is so redundant and not always reliable.	1	0.1%
Through loud speakers	1	0.1%
Through speakers in all buildings	1	0.1%
TV	1	0.1%
UF alerts are fine. none	1	0.1%
University officials should have an emergency application on phones to alert students or have sirens or speakers to announce different situations. Students should be more aware of the protocols incase of an emergency.	1	0.1%
unknown	1	0.1%
Unsure	1	0.1%
UPD drive around with an alert playing on the loud speaker of their cars. That is, if the emergency is serious enough to warrant that.	1	0.1%
updates on situation	1	0.1%
Use more texts/emails	1	0.1%
Use of TV's	1	0.1%
use the loudspeakers	1	0.1%
use the speakers to a better extent.	1	0.1%
Using loud speakers to announce emergency situations.	1	0.1%
Using the TVs in various locations (Reitz, Journalism building, etc.)	1	0.1%

Voice mail	1	0.1%
What steps to take in case of an emergency.	1	0.1%

Table 5

Raw responses to: How could the University of Florida improve the UF Alert system?

Response	*n*	%
MISSING	191	25.6%
Be more timely about the situations that occur	1	0.1%
A little bit more clear on details	1	0.1%
a little more information.	1	0.1%
A lot of the texts I have received have talked about potential threats and in locations that are not close to me so I usually disregard it. If it was an actual emergency, it would be beneficial to have a link to what I need to do next to gain further information.	1	0.1%
Actually focus on the "crime" or threat that is occuring around campus, rather than focus on giving out tickets all day. A man escaped from a police car handcuffed and was not found until 11 hours later, but UPD and all of the other service people were giving tickets like a field day.	1	0.1%
Add a UF alert on local TV	1	0.1%
Add information about what you should for safety precautions instead of just what is happening. Tell me what I should do to avoid or if i get near the situation	1	0.1%
Alerting about incidents that will affect the students most.	1	0.1%
all info at once not multiple ones	1	0.1%
allow texting back	1	0.1%
announce on loud speakers	1	0.1%
As long as it's fast, efficient and tells the area of the incident and what to do, the alert system should be extremely effective.	1	0.1%
Be a little bit more detailed with whom they are trying to get into custody ex: looks, what they are wearing, if they are traveling by themselves	1	0.1%
be a little more clear in the UF alerts, they are sometimes confusing.	1	0.1%
Be a little more timely	1	0.1%
Be clear, concise, informative. No extra info.	1	0.1%

Be clearer in its messages. Not send redundant messages	1	0.1%
Be clearer in some of the descriptions. Many of them are too vague, and narrow down the population of interest to a pretty large group of people--for example, "black, male, with dreds wearing a white t-shirt" describes a significant portion of the black, male population on any given day. It allows for a lot of racial profiling. Consolidate the messages to include as little, but as direct, information as possible: location, crime/suspicion, description, what we as students should do. Use abbreviations where appropriate and be consistent ("unkn" = "unknown" is not as recognizable as "info" = "information").	1	0.1%
Be faster	1	0.1%
Be less racist! Stop including race. Make the announcement more on details other than race.	1	0.1%
Be more accurate & descriptive	1	0.1%
Be more clear	1	0.1%
Be more clear and concise in the information they provide.	1	0.1%
Be more clear and detailed	1	0.1%
Be more clear on where the danger is.	1	0.1%
Be more cohesive and less vague in the texts that are sent out	1	0.1%
be more descriptive	1	0.1%
Be more descriptive	1	0.1%
Be more descriptive about the suspect	1	0.1%
Be more descriptive. And not send out alerts that say that you have not caught him still an hour later.	1	0.1%
Be more detailed in their description of the incident	1	0.1%
Be more detailed, and timely	1	0.1%
be more detailed, don't use acronyms.	1	0.1%
Be more informative about what is going on and more updates as it is going on	1	0.1%

Be more informative and DETAILED.	1	0.1%
Be more informative and more clear/detailed..Just telling us what they are wearing is not very helpful..they could change their clothes anytime..It is too broad	1	0.1%
Be more informative with more details and with more methods of action to take	1	0.1%
Be more specific and less confusing	1	0.1%
Be more specific, provide more details about the situation through a link.	1	0.1%
Be more specific.	2	0.3%
Be more timely	1	0.1%
Be more timely and more clear with descriptions	1	0.1%
be more timely. Half the time we hear outdated info.	1	0.1%
Being more clear	1	0.1%
Being more timely	1	0.1%
better description of incident	1	0.1%
Better sentence structure. I know there is a text message letter limit, but it could be a little more clear.	1	0.1%
By making the alert system more detailed. Instead of stating that a person is still on the run, state specifically where they were seen last.	1	0.1%
By increasing the speed in which we receive the text messages.	1	0.1%
By making it more straightforward.	1	0.1%
by making the messages more concise.	1	0.1%
By making them more timely. It's also a bit unnerving when the alert ends with the suspect not being apprehended by the police.	1	0.1%
by not being vague	1	0.1%
By providing more accurate descriptions of ALL the assailants. Not only the one that is of color.	1	0.1%
By providing more detailed reports and sending a link to a map of the campus for reference, and possibly even highlight the area under alert on the provided map.	1	0.1%

By providing more information with each alert and including a way to respond to each alert	1	0.1%
By releasing the info in a more timely manner	1	0.1%
By sending out alerts on a more timely fashion in regards to updates.	1	0.1%
By using more clear terms.	1	0.1%
Cant	1	0.1%
catch the people	1	0.1%
clear/ more details on what is happening	1	0.1%
Clearer and sooner	1	0.1%
Clearer descriptions	1	0.1%
clearer messages	1	0.1%
Clearer messages -- some of the abbreviations make it difficult to understand. Also, provide tips for what to do during the situation that has arisen.	1	0.1%
clearer messages, don't repeat information	1	0.1%
clearer messages, no abbreviations	1	0.1%
configure a more time efficient computer program to send out messages	1	0.1%
Consolidate alerts into one or two messages	1	0.1%
Continue what they are doing.	1	0.1%
could be sent out quicker w/ more information	1	0.1%
create an app and encourages all student faculty and staff to download thorugh excessive advertisement.	1	0.1%
Details	1	0.1%
Develop protocols in different scenarios so that students are not unaware of what to do when they receive an alert. Stress to students how important it is to not ignore the alerts.	1	0.1%
digital signs around campus	1	0.1%
Do a PA system/similar in Turlington or Plaza of the Americas	1	0.1%
Do not delay messages	1	0.1%

do not email and text me numerous times. send me one text to let me know the situation and know what to do. Then let me know when i can return to normal activity	1	0.1%
Do not use such weird words. Maybe call and leave a message on my phone. More detailed emails	1	0.1%
Don't abbreviate as much	1	0.1%
Don't send the same message multiple times	1	0.1%
Don't send them multiple times	1	0.1%
dont know	1	0.1%
dont send out the alerts that are just a test	1	0.1%
dont send repetitive info	1	0.1%
Educate students on the warning system itself and allow them to choose a method of warning.	1	0.1%
Emailing is not the most efficient way to communicate alerts, but the cellphones is the best way because everybody if non a big percentage of students have cellphones and the messages can get around quicker. Also having a color coded light that flashes in buildings can also make it easier to get the alerts thru campus.	1	0.1%
ensure everyone gets an email	1	0.1%
Explain more how to use the system.	1	0.1%
Explain what we should do for each specific emergency	1	0.1%
Facebook	1	0.1%
Faster alerts	1	0.1%
faster response	1	0.1%
Find more efficient ways to send out information faster and more widespread	1	0.1%
Fix and make more of the blue light emergency stations.	1	0.1%
frequent updates	1	0.1%
Give a little more information	1	0.1%
give directions as to what we should do about the alert (ex. evacuate campus, etc.)	1	0.1%
Give instructions on how to react to certain alerts	1	0.1%

Give more detail on how to proceed rather than just what is happening.	1	0.1%
give more details	1	0.1%
Give more details	1	0.1%
give more details in the message	1	0.1%
give more information	1	0.1%
Give suggested instructions i.e. it is suggested students stay indoors, it is suggested that students evacuate the area, basically give more information about what to do.	1	0.1%
Giving us better directions to do under each situation	1	0.1%
have an app for the entire alert having a more detailed description is nice.	1	0.1%
have an app that has detailed updates, rather than a brief text message that isn't always helpful	1	0.1%
have an information page to learn how to use it or a short course to take	1	0.1%
Have an UF alerts app for the smart phones	1	0.1%
Have more detail in alerts so I really understand what's going on. Don't abbreviate too much.	1	0.1%
Have more detail on what and where exactly the event is taking place	1	0.1%
Helpful safety tips	1	0.1%
I already think it's efficient.	1	0.1%
I am content with how it is	1	0.1%
I am unsure - I live in another country and this does not apply to me.	1	0.1%
I believe it is accurate in all its various ways.	1	0.1%
I believe it is already working at an optimal rate.	1	0.1%
I believe that there are no changes necessary.	1	0.1%
I bellieve there is little room for improvement. The system in place is very effective.	1	0.1%
I can't think of any improvements that would help	1	0.1%
I cant think of anything that would improve the UF Alert system.	1	0.1%

I do not know of any way to improve it. Texts definitely are the best way to inform me.	1	0.1%
i do not receive text messages so adding that	1	0.1%
I don't know	3	0.4%
I don't know.	2	0.3%
I don't read the UF Alerts	1	0.1%
i dont know	2	0.3%
I dont know	2	0.3%
I feel as if the UF Alert system is extremely good as it is.	1	0.1%
I like how informative and clear they are, I wouldn't change anything about them.	1	0.1%
I like it	1	0.1%
I like the system they have now	1	0.1%
i never hear updates after the fact. like if an incident occurs on campus and they catch the person the next day, even though its a new day, it'd still be nice to know the status of the incident.	1	0.1%
I never knew that you could respond or interact with the alert system. Having a way of receiving instructions for the action needed following the emergency would be helpful.	1	0.1%
I really don't know	1	0.1%
I take all my courses online so it is hard for me to judge what would be the best option.	1	0.1%
I think it is as good as it can get right now.	1	0.1%
I think it is doing a good job so far.	1	0.1%
I think it is efficient enough currently	1	0.1%
I think it is fine the way it is.	1	0.1%
I think it is good as it is because as I do not live on campus some information that I do recieve is useful.	1	0.1%
I think it is good the way it is	1	0.1%
I think it is good.	1	0.1%
I think it is pretty informative as it is.	1	0.1%
I think it is pretty up to par.	1	0.1%
I think it serves it's purpose.	1	0.1%

i think it's done well	1	0.1%
I think it's fine the way it is	1	0.1%
I think it's good enough	1	0.1%
I think it's pretty spot on right now	1	0.1%
i think its basically fine maybe if its a serious threat tell us what we should do to respond like a fire where we should go	1	0.1%
I think its pretty effective and informative.	1	0.1%
I think its pretty good	1	0.1%
I think that the UF alerts do a good job of telling students what is going on in as timely of a fashion as possible.	1	0.1%
I think the system is good. No need to fix what isn't broken.	1	0.1%
I think the system is solid as is. Especially in comparison to my previous college.	1	0.1%
I think the system should be discussed more to freshman and all students because some students are unaware it is in place.	1	0.1%
I think the system works fine as it is.	1	0.1%
I think the texting alert system is sufficient	1	0.1%
I think they are doing everything they can by the use of every social network or communication device to warn us.	1	0.1%
I understand that the text alerts need to be short and to the point but they should provide a source where a more detailed report of what is going on can be found such as a website, or something. There, they should include what to do according to the situation.	1	0.1%
I'm not sure at this point	1	0.1%
IDL	1	0.1%
If you do not know what exactly is going on, do not send them. I remember one night I got an alert saying there was a suspect near rawlings and it turned out not to be a suspect at all and I was scared out of my mind.	1	0.1%

Important messages could be sent as soon as an emergency event occurs and follow-up information could be condensed into one or two text messages instead of several messages within a short time span that basically relay the same information.	1	0.1%
Improve clarity of messages	1	0.1%
Improve Gville PD and actually catch the suspects instead of alerting us that they were never found.	1	0.1%
Improve the grammar/abbreviations	1	0.1%
Improve the wording of the messages	1	0.1%
Improve wording..	1	0.1%
In the message they should tell students what they should do about the situation. Where to go.	1	0.1%
include announcements in libraries and on campus buildings	1	0.1%
include appropriate response	1	0.1%
Include clearer instructions on what to in certain emergency situations	1	0.1%
Include instructions on what to do if there is a gunman on campus, inclement wether, or any other serious issue	1	0.1%
Include tips and detailed information. A reply for more information service	1	0.1%
Including how to respond to the alert, not just the alert itself.	1	0.1%
Inform more people about it. Maybe install a PA/speaker system throughout campus, however it may be distracting.	1	0.1%
Inform students how to get alerts via text message and make the notices more clear	1	0.1%
Inform students when the suspect has been apprehended not just inform us that they are on the loose.	1	0.1%
Informing on how to react	1	0.1%
It could maybe be more specific of places to avoid and what to do in such emergency situations.	1	0.1%

it is already good	1	0.1%
It is fine just how it is.	1	0.1%
it is fine the way it is	1	0.1%
It is fine.	1	0.1%
it is good	1	0.1%
it is good as it is	1	0.1%
It is good enough.	1	0.1%
it is ok	1	0.1%
it is pretty good	1	0.1%
It is very effective.	1	0.1%
It seems fine	1	0.1%
It seems perfect the way it is.	1	0.1%
It seems very effective. I can't think of anything that would improve it.	1	0.1%
It should be as clear as possible	1	0.1%
It's fine as is	1	0.1%
It's fine now.	1	0.1%
It's fine.	1	0.1%
It's good as it is.	1	0.1%
it's good the way it is	1	0.1%
It's good.	1	0.1%
It's really annoying when it keeps mentioning all the people that aren't caught.	1	0.1%
its fine how it is	1	0.1%
Its good already	1	0.1%
Its good as is	1	0.1%
Just make sure that it is more clear sometimes I read them and I don't really understand the abbreviations.	1	0.1%
just make the text messages a little more informative and clear. the general assessment of the situations seem to just be repeats of text messages sent in the past	1	0.1%
Less abbreviations	1	0.1%
less abbreviations in the text messages. otherwise, pretty helpful	1	0.1%
less annoying	1	0.1%
less confusing texts	1	0.1%

less frequent; only send most important stuff	1	0.1%
Less redunancy	1	0.1%
less redundant	1	0.1%
Less redundant text messages, more informative messages.	1	0.1%
less texts	1	0.1%
less unnecessary texts, only vital information	1	0.1%
less vague	1	0.1%
Let people know how to respond.	1	0.1%
Let us know more details and what to do	1	0.1%
Letting students know it is on facebook and also on twitter	1	0.1%
Limit the amount of alerts sent	1	0.1%
Link in message for how to respond to various threats/alerts.	1	0.1%
Loud speakers and digital billboards	1	0.1%
make an app	1	0.1%
Make it easier to resond to and more clear	1	0.1%
make it easier to use	1	0.1%
make it faster and less alerts	1	0.1%
make it faster? not sure if go out as somethings happening..	1	0.1%
Make it less vague, if there is an alert have it placed on ISIS as well or any of the main computers on campus. Have them make announcements to those in Lib West or Martson, etc.	1	0.1%
Make it more clear and make sure it is efficient and accurate	1	0.1%
make it more clear, and easier to understand. Don't use law inforcement terms	1	0.1%
Make it more clear, too vague and confusing to understand shorthand at times	1	0.1%
make it more concise and stop sending multiple nessages	1	0.1%
Make it more descriptive	1	0.1%
make it more detailed and give us information on how to respond to information given	1	0.1%

Make it more efficient by sending out the texts, emails, etc, sooner. Also, i do not always get the 2nd text of a message so imporoving their system to make sure the whole text is getting sent to ppl	1	0.1%
Make it more efficient, and more informative	1	0.1%
Make it quicker	1	0.1%
make it where we know what is being said. some of the abbreviations are not straight forward and we are guessing sometimes.	1	0.1%
Make messages more clear. I don't like to read text messages written in abbreviations	1	0.1%
make more clear	1	0.1%
Make signing up for text message notifications mandatory and very easy to do. Put it on ISIS and make students have holds on their accounts unless they sign up for a form of alert notification. But make the signing up process simple and easy to do/find.	1	0.1%
Make sure everyone updates their contact information so that everyone can be informed.	1	0.1%
make sure everyone updates their phone numbers	1	0.1%
make sure it is fast	1	0.1%
make sure that it goes to every student's phone, make the messages less vague	1	0.1%
make sure the information is more than 97% accurate before sending it out.	1	0.1%
make sure the text message system is working	1	0.1%
Make text message more coherent, no abbreviations	1	0.1%
Make texts less vague, and more detailed	1	0.1%
make the alerts come out as fast as possible	1	0.1%
Make the alerts more clear because a lot of times they are extremely hard to figure out what is going on. Whoever abbreviates words/writes the alerts does a horrible job	1	0.1%
Make the alerts more detailed. Add more incidents, such as car crashes etc.	1	0.1%

Make the description more descriptive.	1	0.1%
Make the messages a little more detailed. (More content)	1	0.1%
make the messages clearer.	1	0.1%
make the messages for clear	1	0.1%
make the messages more clear	1	0.1%
Make the messages more clear and concise and follow up the next day or when the person or threat has been cleared.	1	0.1%
make the messages more clear and less redundant	1	0.1%
Make the messages more detailed	1	0.1%
make the messages more informative	1	0.1%
make the text messages a little more clear	1	0.1%
Make the text messages clearer, sometimes they can be vague and I am not clear what exactly is happening.	1	0.1%
Make the texts a little bit more detailed	1	0.1%
Make the texts less vague	1	0.1%
Make the texts more clear	1	0.1%
Make the texts more specific and tell you the best plan of action to take.	1	0.1%
Make the UF alerts more elaborate. The term "B/M" has been used, and I think that's a bad abbreviation for black male". Give more details about what students should do, and ALWAYS follow up with updates/resolutions.	1	0.1%
Make them clear and concise. The average person does not understand certain police jargon such as "strong hand"	1	0.1%
Make them more clear	1	0.1%
make them more clear and include how students should respond	1	0.1%
Make them more clear on what the danger is relative to UF and provide information on how to take action.	1	0.1%
Make them more consistent	1	0.1%
make them more detailed	1	0.1%

Making it more clear on what students should do to avoid that particular danger	1	0.1%
Making it more easier to understand the alerts.	1	0.1%
Making the messages a little more clear and not sending the messages multiple times saying the same thing. Also keep the number that the alerts are coming from consistent. I will get texts from two different numbers sometimes and one will give the initial update then the other will give a follow up and they go back and forth. It gets confusing.	1	0.1%
making the text messages more detailed and using proper words rather than abbreviations for everything.	1	0.1%
maybe being more detailed?	1	0.1%
Maybe include incidents that happen off campus, but close enough to it that students should know.	1	0.1%
Maybe not use so much short hand. For example, "UF Alert Susp still at large left in unk direction..." I assume that "unk" means unknown? Just small things like that, but overall I think you guys do a great job!	1	0.1%
Maybe show pics of the subjects when located	1	0.1%
Maybe with a clearer message because the messages are usually vague, irrelevant and there is never anything I can do about it.	1	0.1%
megaphone	1	0.1%
monitor efficiency, reliability, and usefulness.	1	0.1%
more accurate messages, update only on important and vital statuses	1	0.1%
More characters in msg	1	0.1%
more clarity	1	0.1%
more clear	1	0.1%
More clear	1	0.1%
More clear text messages	1	0.1%
More clear updates	1	0.1%
more descriptive	2	0.3%
More descriptive	3	0.4%

More descriptive details and doesn't bombard my phone with a whole bunch of texts and emails repeating the same thing	1	0.1%
More descriptive explanations	1	0.1%
More descriptive in what has actually happened and have the message sent out in a manner that is more swift than it currently is.	1	0.1%
more detail	3	0.4%
More detail	1	0.1%
More detailed	2	0.3%
More detailed alerts	1	0.1%
More detailed and clear	1	0.1%
more detailed and give instruction on acttion	1	0.1%
more detailed and meaningful	1	0.1%
More detailed and timely alerts	1	0.1%
more detailed descriptions	1	0.1%
more detailed evacuation plan	1	0.1%
more detailed information	1	0.1%
More detailed information	1	0.1%
more detailed locations	1	0.1%
more detailed messages that are honest	1	0.1%
More detailed text messages.	1	0.1%
more detailed, and describe if we need to do anything to protect ourselves from the incident	1	0.1%
More detailed.	1	0.1%
More detailed/informative messages	1	0.1%
more details	1	0.1%
More details	2	0.3%
More details and a recommended reponse.	1	0.1%
more details and update more often about dangerous situations	1	0.1%
more details at the time of the incident, more timely warnings as soon as they happen, not delaying the messages to alert students and faculty promptly	1	0.1%
More details of situation. recommended actions to take/avoid, areas to avoid	1	0.1%
more discription of the message	1	0.1%

More frequent updates	1	0.1%
more functions	1	0.1%
more info	1	0.1%
More information about how students shouls react.	1	0.1%
more methods of alerts	1	0.1%
More on time and more description.	1	0.1%
More outlets for emergency situations	1	0.1%
more prompt	1	0.1%
more specfic description over text/email	1	0.1%
More specific and clear	1	0.1%
More specific description of the situation rather than using abbreviations and shorthand.	1	0.1%
More specific details	1	0.1%
More texting	1	0.1%
more texts	1	0.1%
More timely	1	0.1%
More timely and accurate. Provide what to do in case of major emergencies (Code Black, Red, Silver, etc)	1	0.1%
More timely texts	1	0.1%
More timely.	1	0.1%
more updates	1	0.1%
More updates and more timely	1	0.1%
n/a	9	1.2%
N/a	1	0.1%
N/A	15	2.0%
NA	1	0.1%
need more blue lights on sorority row	1	0.1%
no comment	2	0.3%
no idea	1	0.1%
No improvement is necessary	1	0.1%
No improvements	1	0.1%
no repetitive message... ways to evacuate or places to avoid	1	0.1%
No suggestions	1	0.1%
NO SUGGESTIONS	1	0.1%
no way	1	0.1%

No way	1	0.1%
none	7	0.9%
None	5	0.7%
None.	2	0.3%
Not be so redundant and send so many alerts about the same thing... people will stop looking at the alerts	1	0.1%
not really sure, its pretty good	1	0.1%
not send 10 text messages about the situation	1	0.1%
Not send out notification for things after they have already been dealt with.	1	0.1%
Not send out so many, only the important and urgent announcements	1	0.1%
Not send so many redundant and repetitive messages. I understand they're important, but there's a suspicious man and I receive 8 text messsages and 8 e-mails. It's a bit overkill.	1	0.1%
not send so many texts about the same situation	1	0.1%
not sending as many texts	1	0.1%
not so many alerts	1	0.1%
not such short texts. rather get a good text of information than 3 word phrases that don't always make sense.	1	0.1%
not sure	5	0.7%
Not sure	2	0.3%
Not sure, don't completely understand it	1	0.1%
Not sure. The texts are somewhat annoying if I'm not on campus but they are very useful when I am. I think the benefits are much greater than the slight annoyance.	1	0.1%
not use as many abbreviations/be more descriptive	1	0.1%
nothing	1	0.1%
nothing it is fine the way it is	1	0.1%
Nothing. It is really good already.	1	0.1%
Notify students in specific areas of Gainesville if something happens in their area of dwelling	1	0.1%

Only send alerts to people who live within a living distance that may legitimately stand to benefit from the information.	1	0.1%
only send out information that is necessary	1	0.1%
Perhaps, advise students the severity of the situation and provide tips on how to remain safe.	1	0.1%
Please provide more detailed information of exact location of occurrences and what to do.	1	0.1%
Possibly make the alerts more descriptive	1	0.1%
Proof messages before sending, keep more direct and straight forward	1	0.1%
Provide a solution when stating an issue.	1	0.1%
provide a text that says what we should do in response to the situation	1	0.1%
Provide actions that we as students need to take in the event of evacuations or threats	1	0.1%
provide acurate, detailed information	1	0.1%
Provide more detail	1	0.1%
Provide more detailed descriptions as well as clarifying locations on campus.	1	0.1%
Provide more detailed descriptions of the event.	1	0.1%
Provide more detailed information and what students should do	1	0.1%
provide more details on subject's appearance (if available)	1	0.1%
Provide more details. The alerts are very vague.	1	0.1%
provide more helpful info about how to react/ help/ escape	1	0.1%
provide more information about location of problems, include which areas of campus are at high risk	1	0.1%
provide more information in the text message, put alerts on facebook	1	0.1%
provide more information in the texts they send out	1	0.1%
provide one alert until significant changes occur. less redundant updates	1	0.1%

provide suggestions as to how to react to each alert. ex: stay on campus, leave campus, avoid a certain route	1	0.1%
Provide suggestions on how to handle the information	1	0.1%
provide us with information on how to handle specific situatuions	1	0.1%
providing audio notification	1	0.1%
Providing instructions for what we are supposed to do when we get an alert that may affect us.	1	0.1%
providing more details	1	0.1%
quicker messages. I once got a message the next day on my email that I was one block away from an incident the night before and had had no idea	1	0.1%
quicker response	1	0.1%
quicker response times after incident.	1	0.1%
Rate how serious the crime being reported is	1	0.1%
Refrain from using abbreviations and provide more detail/leave out unimportant details. Ex. When I read the most recent alert I did not know what "unk" race stood for. Also, if UF does not know what race the subject is, why should they even include that in the message?	1	0.1%
Release the information as quickly as possible. Students need to be aware	1	0.1%
Report crimes involving suspects of all races	1	0.1%
report incidients quicker	1	0.1%
require every student has one form of emergency contact information with university	1	0.1%
see last comments	1	0.1%
seems fine	1	0.1%
Seems to be as if it is very racially biased.	1	0.1%
Send alerts with instructions on how to protect yourself in addition to information on the threat.	1	0.1%
send all as one text message	1	0.1%

send better updates of what is going on. i feel like i always get a text of what the problem is but never find out if the problem is resolved. essentially, the uf alerts do not make me feel any safer or more protected	1	0.1%
send emails, texts, and other ways so that all students are informed	1	0.1%
Send fewer alerts. I get so many between email and texting that I start to ignore them.	1	0.1%
Send information about emergencies quicker	1	0.1%
Send information when you have definite information to give. It just seems like I get a bunch of text messages that are kind of unsure about what's going on. Doesn't give any information on how I should handle the situation. Like stay inside or go home etc	1	0.1%
Send it through text messaging and twitter	1	0.1%
Send less text messages or try to condense the texts	1	0.1%
Send messages out sooner.	1	0.1%
send more frequently	1	0.1%
Send more informative text messages by including more information and suggested plan of action. Also using correct grammar.	1	0.1%
send out a map of emergency or crimes location	1	0.1%
Send out alerts sooner, more detail, response information if applicable.	1	0.1%
Send out more emails	1	0.1%
send out more than one email or text	1	0.1%
Send out single messages instead of five parts	1	0.1%
Send out the details of an incident in a more timely manner; provide more details of (for example) suspects of crimes	1	0.1%
send texts that are only 1 page long so that the texts are not too long and confusing to read	1	0.1%
Send the alert when the incident happens, not hours after	1	0.1%
send the alerts throgh on campus speakers	1	0.1%

Send them out faster. Include follow up or how to respond to incident if you are near by.	1	0.1%
send them out when the emergency is taking place so we know about any dangers and where to stay clear of	1	0.1%
shorter more to the point messages	1	0.1%
Single page text messages with no abbreviations. Possibly allow a feature to texting questions in addition to the calling.	1	0.1%
Slghtly more detail	1	0.1%
slightly more detailed reports	1	0.1%
Some of the descriptions seem to be repetitive	1	0.1%
Some people do not recieve the texts until about 15 minutes after I do, so more timely. Sending something out on facebook would also be a very good idea, using a speaker system through buildings, and having RAs post on the floor facebook pages.	1	0.1%
sometimes the descriptions are very vague so maybe being a little more descriptive. I also think that some of the updates are kind of useless like when they say nothing really has changed.	1	0.1%
sometimes the messages get cut off which makes it harder to get the entire alert. fix that problem some how	1	0.1%
Sometimes the words aren't ordered clearly or words are left out so the message is confusing. Otherwise it's pretty straightforward. Maybe with more personalized instructions on what to do depending on where you are.	1	0.1%
Sometimes there's delayed information. Also, sometimes the abbreviations are unclear	1	0.1%
speaker system	1	0.1%
Speed of texts	1	0.1%
stop abbreviating things	1	0.1%
Stop being so vague and send more regular updates.	1	0.1%

Stop sending both text messages and emails. Should only be sent to email. Texts get annoying and disruptive	1	0.1%
Stop the redundancy.	1	0.1%
stop using confusing abbreviations	1	0.1%
survey	1	0.1%
Take advantage of location GPS services to pinpoint where its happening if students wanted to, also inform students more on how to respond and encourage it more	1	0.1%
tell us what to do, should we be in the middle of the situation	1	0.1%
Tell us what we should do to be safe	1	0.1%
Text instead of email	1	0.1%
Text me earlier	1	0.1%
Text messages sent when something occurs	1	0.1%
texting	1	0.1%
texts	1	0.1%
Texts are often not too clear since it contains many abbreviations and tries to save space. Perhaps, have a website that contains more detailed information where text message receivers can further inform themselves.	1	0.1%
The alert system seems pretty good, I can't see a way to improve it. Texting is definitely efficient and quick and everyone checks their phone.	1	0.1%
The descirptions usually can describe half of campus. More details.	1	0.1%
The students can know more about it. I don't even know the number to call. I also do not know what to do with the emergency buttons on campus.	1	0.1%
The text messages are not worded that well. They are also very redundant.	1	0.1%
The text messages are useful and beneficial.	1	0.1%
the texts should be more detailed	1	0.1%

The UF alert system could be improved by having everyone subscribe to it and provide meetings or seminars on how to protect and react to a situation such as a gunman or extreme weather.	1	0.1%
The UF alert system is very effective in my opinion. I think the best way to improve the system is to make them even more timely and more specific with locations on campus.	1	0.1%
There is a lot of repetitive messages, especially when they are broken up into multiple texts. Can be annoying when they say essentially the same thing. Maybe state that you should avoid the area depending on the situation and let police know if you have information, but continual texts are redundant	1	0.1%
There is no way.	1	0.1%
They are doing a pretty good job.	1	0.1%
They cannot.	1	0.1%
They could be more specific.	1	0.1%
timely, more descriptive	1	0.1%
to have somebody that people communicate in person	1	0.1%
update earlier	1	0.1%
use a better texting system so they dont have to use so much slang and can write more- that way itll be easier to understand	1	0.1%
use clearer language and provide protection instructions	1	0.1%
Use code words so that if speaker systems are used, at least the culprit won't know what's going on	1	0.1%
Use digital billboards to update students on events	1	0.1%
Use fluent sentences in texts	1	0.1%
use less abbreviations in the uf alerts, they are sometimes confusing	1	0.1%
Use proper grammar and punctuation and avoid abbreviations when possible	1	0.1%

Using Phone Calls	1	0.1%
Usually I get the UF Alert texts about an hour after the event happened, so if I was walking into a dangerous situation on or off campus, I wouldn't know until later. If texts could somehow be sent out quicker, I would feel safer knowing that I am getting information as soon as possible. Students will always complain about texts, but I think it is the most effective method to alert people.	1	0.1%
utilize PA's	1	0.1%
Videos, pictures, and or articles	1	0.1%
What to do	1	0.1%
with each alert, send an email describing what actions should be taken if you are on campus or near the area where the incident occurred	1	0.1%
Works fine in my opinion	1	0.1%
Write more clear messages because they are often confusing	1	0.1%
