# Supplementary material for: When minutes matter: A university emergency notification system dataset
Source: Data Brief. 2021 Feb 26;35:106910. doi: 10.1016/j.dib.2021.106910 (PMC8010384; doi:10.1016/j.dib.2021.106910)
Supplement: Supplementary file 4 [file mmc4.pdf]

Codebook for *When Minutes Matter: A University Emergency Notification System Dataset by Menn, Payne-Purvis, Chaney, and Chaney*

File Information

Number of Cases	Unweighted	746
	Weighted	746

informedconsent

Value	Count	Percent
Begin survey (I consent to participating in this study)	746	100.0%
I do not want to participate in this study	0	0.0%
I have already participated in this study	0	0.0%

term

		Value	Count	Percent
Standard Attributes	Label	Term data were collected		
Valid Values	1	Fall 2012	410	55.0%
	2	Spring 2013	273	36.6%
	3	Summer 2013	63	8.4%

receivedreadone

		Value	Count	Percent
Standard Attributes	Label	Have you received and/or read at least one UF Alert message since you have been a student at UF?		
Valid Values	1	Yes	738	98.9%
	2	No	6	0.8%
	3	I am not a student at the University of Florida	0	0.0%
Missing Values	System		2	0.3%

receivedemail

		Value	Count	Percent
Standard Attributes	Label	Have you received at least one UF Alert message through the following channels? - Email		
Valid Values	1	Yes	700	93.8%
	2	No	33	4.4%
Missing Values	System		13	1.7%

receivedtext

		Value	Count	Percent
Standard Attributes	Label	Have you received at least one UF Alert message through the following channels? - Text message		
Valid Values	1	Yes	675	90.5%
	2	No	60	8.0%
Missing Values	System		11	1.5%

receivedtwitter

		Value	Count	Percent
Standard Attributes	Label	Have you received at least one UF Alert message through the following channels? - Twitter		
Valid Values	1	Yes	55	7.4%
	2	No	645	86.5%
Missing Values	System		46	6.2%

receivedfacebook

		Value	Count	Percent
Standard Attributes	Label	Have you received at least one UF Alert message through the following channels? - Facebook		
Valid Values	1	Yes	58	7.8%
	2	No	642	86.1%
Missing Values	System		46	6.2%

receivedclassroom

		Value	Count	Percent
Standard Attributes	Label	Have you received at least one UF Alert message through the following channels? - Classroom or laboratory telephone or speaker system		
Valid Values	1	Yes	37	5.0%
	2	No	671	89.9%
Missing Values	System		38	5.1%

receivedUFwebpage

		Value	Count	Percent
Standard Attributes	Label	Have you received at least one UF Alert message through the following channels? - University of Florida webpage		
Valid Values	1	Yes	140	18.8%
	2	No	568	76.1%
Missing Values	System		38	5.1%

receivedRSS

		Value	Count	Percent
Standard Attributes	Label	Have you received at least one UF Alert message through the following channels? - RSS Feed		
Valid Values	1	Yes	36	4.8%
	2	No	672	90.1%
Missing Values	System		38	5.1%

receivedoutdoorturlington

		Value	Count	Percent
Standard Attributes	Label	Have you received at least one UF Alert message through the following channels? - Outdoor speakers in Turlington Plaza		
Valid Values	1	Yes	15	2.0%
	2	No	309	41.4%
Missing Values	System		422	56.6%

receivedoutdoorplaza

		Value	Count	Percent
Standard Attributes	Label	Have you received at least one UF Alert message through the following channels? - Outdoor speakers in the Plaza of the Americas		
Valid Values	1	Yes	12	1.6%
	2	No	312	41.8%
Missing Values	System		422	56.6%

receivedoutdoorsun

		Value	Count	Percent
Standard Attributes	Label	Have you received at least one UF Alert message through the following channels? - Outdoor speakers in The Sun Terrace		
Valid Values	1	Yes	6	0.8%
	2	No	316	42.4%
Missing Values	System		424	56.8%

receivedoutdoorreitz

		Value	Count	Percent
Standard Attributes	Label	Have you received at least one UF Alert message through the following channels? - Outdoor speakers around the Reitz Student Union lawn		
Valid Values	1	Yes	12	1.6%
	2	No	312	41.8%
Missing Values	System		422	56.6%

receivedhistorical1

		Value	Count	Percent
Standard Attributes	Label	Did you receive any of the following UF Alerts? - UF Alert Ref armed subject Shands Gainesville, Garage10. Officers clearing location. Please refer to http://www.ufl.edu for details.		
Valid Values	1	Yes	504	67.6%
	2	No	60	8.0%
	3	I'm not sure	174	23.3%
Missing Values	System		8	1.1%

receivedhistorical2

		Value	Count	Percent
Standard Attributes	Label	Did you receive any of the following UF Alerts? - (1 of 2) UF Alert Ref incident at Shands Gnvl in Garage10 armed Black male subject believed to have left the campus area Call 352-392-1111		
Valid Values	1	Yes	495	66.4%
	2	No	67	9.0%
	3	I'm not sure	176	23.6%
Missing Values	System		8	1.1%

receivedhistorical3

		Value	Count	Percent
Standard Attributes	Label	Did you receive any of the following UF Alerts? - (2 of 2) with info 2 of 3 message		
Valid Values	1	Yes	440	59.0%
	2	No	70	9.4%
	3	I'm not sure	220	29.5%
Missing Values	System		16	2.1%

receivedhistorical4

		Value	Count	Percent
Standard Attributes	Label	Did you receive any of the following UF Alerts? - (1 of 2) UF Alert Reference armed disturbance reported at Shands Gainesville in Garage 10. Two subjects, one black male armed with handgun.		
Valid Values	1	Yes	475	63.7%
	2	No	65	8.7%
	3	I'm not sure	196	26.3%
Missing Values	System		10	1.3%

receivedhistorical5

		Value	Count	Percent
Standard Attributes	Label	Did you receive any of the following UF Alerts? - (2 of 2) 1 of 2 messages		
Valid Values	1	Yes	427	57.2%
	2	No	76	10.2%
	3	I'm not sure	219	29.4%
Missing Values	System		24	3.2%

receivedhistorical6

		Value	Count	Percent
Standard Attributes	Label	Did you receive any of the following UF Alerts? - UF Alert Location of incident is Gar10 Shands Gainesville no injuries reported at this time.		
Valid Values	1	Yes	399	53.5%
	2	No	89	11.9%
	3	I'm not sure	244	32.7%
Missing Values	System		14	1.9%

receivedhistorical7

		Value	Count	Percent
Standard Attributes	Label	Did you receive any of the following UF Alerts? - UF Alert 2 subjects detained, 1 black male white shirt with hand gun at large. Report info to 352-392-1111		
Valid Values	1	Yes	453	60.7%
	2	No	66	8.8%
	3	I'm not sure	217	29.1%
Missing Values	System		10	1.3%

receivedhistorical8

		Value	Count	Percent
Standard Attributes	Label	Did you receive any of the following UF Alerts? - UF Alert Reported armed 2 males subj's unk. race at shands Gar.10 unknown intent. UPD on scene. Report info to 352-392-1111		
Valid Values	1	Yes	422	56.6%
	2	No	81	10.9%
	3	I'm not sure	234	31.4%
Missing Values	System		9	1.2%

receivedhistorical9

		Value	Count	Percent
Standard Attributes	Label	Did you receive any of the following UF Alerts? - UF Alert Reference the Bomb Threat at Shands UF the threat was a hoax and two are now in custody.		
Valid Values	1	Yes	481	64.5%
	2	No	83	11.1%
	3	I'm not sure	174	23.3%
Missing Values	System		8	1.1%

receivedhistorical10

		Value	Count	Percent
Standard Attributes	Label	Did you receive any of the following UF Alerts? - UF Alert Shands main campus No suspicious devices located Officers are clearing the area Call 352 392-1111 with any information		
Valid Values	1	Yes	456	61.1%
	2	No	84	11.3%
	3	I'm not sure	196	26.3%
Missing Values	System		10	1.3%

respond1

		Value	Count	Percent
Standard Attributes	Label	Did you respond to any of the following UF Alerts? (By respond we mean did you take any actions such as leaving campus, evacuating a building, avoiding an area of campus, contacting a law enforcement agency, or alerting other individuals to the notification). - UF Alert Ref armed subject Shands Gainesville, Garage10. Officers clearing location. Please refer to http://www.ufl.edu for details.		
Valid Values	1	Yes	176	23.6%
	2	No	449	60.2%
	3	No, I did not know how to respond	109	14.6%
Missing Values	System		12	1.6%

respond2

		Value	Count	Percent
Standard Attributes	Label	Did you respond to any of the following UF Alerts? (By respond we mean did you take any actions such as leaving campus, evacuating a building, avoiding an area of campus, contacting a law enforcement agency, or alerting other individuals to the notification). - (1 of 2) UF Alert Ref incident at Shands Gnvl in Garage10 armed Black male subject believed to have left the campus area Call 352-392-1111		
Valid Values	1	Yes	165	22.1%
	2	No	455	61.0%
	3	No, I did not know how to respond	112	15.0%
Missing Values	System		14	1.9%

respond3

		Value	Count	Percent
Standard Attributes	Label	Did you respond to any of the following UF Alerts? (By respond we mean did you take any actions such as leaving campus, evacuating a building, avoiding an area of campus, contacting a law enforcement agency, or alerting other individuals to the notification). - (2 of 2) with info 2 of 3 message		
Valid Values	1	Yes	156	20.9%
	2	No	453	60.7%
	3	No, I did not know how to respond	121	16.2%
Missing Values	System		16	2.1%

respond4

		Value	Count	Percent
Standard Attributes	Label	Did you respond to any of the following UF Alerts? (By respond we mean did you take any actions such as leaving campus, evacuating a building, avoiding an area of campus, contacting a law enforcement agency, or alerting other individuals to the notification). - (1 of 2) UF Alert Reference armed disturbance reported at Shands Gainesville in Garage 10. Two subjects, one black male armed with handgun.		
Valid Values	1	Yes	165	22.1%
	2	No	457	61.3%
	3	No, I did not know how to respond	111	14.9%
Missing Values	System		13	1.7%

respond5

		Value	Count	Percent
Standard Attributes	Label	Did you respond to any of the following UF Alerts? (By respond we mean did you take any actions such as leaving campus, evacuating a building, avoiding an area of campus, contacting a law enforcement agency, or alerting other individuals to the notification). - (2 of 2) 1 of 2 messages		
Valid Values	1	Yes	153	20.5%
	2	No	462	61.9%
	3	No, I did not know how to respond	112	15.0%
Missing Values	System		19	2.5%

respond6

		Value	Count	Percent
Standard Attributes	Label	Did you respond to any of the following UF Alerts? (By respond we mean did you take any actions such as leaving campus, evacuating a building, avoiding an area of campus, contacting a law enforcement agency, or alerting other individuals to the notification). - UF Alert Location of incident is Gar10 Shands Gainesville no injuries reported at this time.		
Valid Values	1	Yes	158	21.2%
	2	No	462	61.9%
	3	No, I did not know how to respond	112	15.0%
Missing Values	System		14	1.9%

respond7

		Value	Count	Percent
Standard Attributes	Label	Did you respond to any of the following UF Alerts? (By respond we mean did you take any actions such as leaving campus, evacuating a building, avoiding an area of campus, contacting a law enforcement agency, or alerting other individuals to the notification). - UF Alert 2 subjects detained, 1 black male white shirt with hand gun at large. Report info to 352-392-1111		
Valid Values	1	Yes	169	22.7%
	2	No	452	60.6%
	3	No, I did not know how to respond	111	14.9%
Missing Values	System		14	1.9%

respond8

		Value	Count	Percent
Standard Attributes	Label	Did you respond to any of the following UF Alerts? (By respond we mean did you take any actions such as leaving campus, evacuating a building, avoiding an area of campus, contacting a law enforcement agency, or alerting other individuals to the notification). - UF Alert Reported armed 2 males subj's unk. race at shands Gar.10 unknown intent. UPD on scene. Report info to 352-392-1111		
Valid Values	1	Yes	158	21.2%
	2	No	457	61.3%
	3	No, I did not know how to respond	119	16.0%
Missing Values	System		12	1.6%

respond9

		Value	Count	Percent
Standard Attributes	Label	Did you respond to any of the following UF Alerts? (By respond we mean did you take any actions such as leaving campus, evacuating a building, avoiding an area of campus, contacting a law enforcement agency, or alerting other individuals to the notification). - UF Alert Reference the Bomb Threat at Shands UF the threat was a hoax and two are now in custody.		
Valid Values	1	Yes	176	23.6%
	2	No	444	59.5%
	3	No, I did not know how to respond	110	14.7%
Missing Values	System		16	2.1%

respond10

		Value	Count	Percent
Standard Attributes	Label	Did you respond to any of the following UF Alerts? (By respond we mean did you take any actions such as leaving campus, evacuating a building, avoiding an area of campus, contacting a law enforcement agency, or alerting other individuals to the notification). - UF Alert Shands main campus No suspicious devices located Officers are clearing the area Call 352 392-1111 with any information		
Valid Values	1	Yes	163	21.8%
	2	No	456	61.1%
	3	No, I did not know how to respond	115	15.4%
Missing Values	System		12	1.6%

respondshelter

		Value	Count	Percent
Standard Attributes	Label	To what extent do you agree or disagree with the following statements? If I received a UF Alert about an on campus _________________________, I would know how to respond. - Shelter-In-Place		
N	Valid	743		
	Missing	3		
Central Tendency and Dispersion	Mean	4.01		
	Standard Deviation	1.859		
Labeled Values	1	Strongly agree	84	11.3%
	2	Moderately agree	92	12.3%
	3	Somewhat agree	110	14.7%
	4	Neutral (neither disagree nor agree)	189	25.3%
	5	Somewhat disagree	89	11.9%
	6	Moderately disagree	74	9.9%
	7	Strongly disagree	105	14.1%

respondevac

		Value	Count	Percent
Standard Attributes	Label	To what extent do you agree or disagree with the following statements? If I received a UF Alert about an on campus __________________________, I would know how to respond. - Building Evacuation		
N	Valid	742		
	Missing	4		
Central Tendency and Dispersion	Mean	2.89		
	Standard Deviation	1.776		
Labeled Values	1	Strongly agree	201	26.9%
	2	Moderately agree	173	23.2%
	3	Somewhat agree	140	18.8%
	4	Neutral (neither disagree nor agree)	91	12.2%
	5	Somewhat disagree	54	7.2%
	6	Moderately disagree	38	5.1%
	7	Strongly disagree	45	6.0%

respondcampusevac

		Value	Count	Percent
Standard Attributes	Label	To what extent do you agree or disagree with the following statements? If I received a UF Alert about an on campus __________________________, I would know how to respond. - Campus Evacuation		
N	Valid	741		
	Missing	5		
Central Tendency and Dispersion	Mean	3.15		
	Standard Deviation	1.875		
Labeled Values	1	Strongly agree	185	24.8%
	2	Moderately agree	139	18.6%
	3	Somewhat agree	140	18.8%
	4	Neutral (neither disagree nor agree)	101	13.5%
	5	Somewhat disagree	69	9.2%
	6	Moderately disagree	50	6.7%
	7	Strongly disagree	57	7.6%

respondchemhazmat

		Value	Count	Percent
Standard Attributes	Label	To what extent do you agree or disagree with the following statements? If I received a UF Alert about an on campus _________________________, I would know how to respond. - Chemical or Hazardous Material Spill		
N	Valid	744		
	Missing	2		
Central Tendency and Dispersion	Mean	3.99		
	Standard Deviation	1.898		
Labeled Values	1	Strongly agree	94	12.6%
	2	Moderately agree	87	11.7%
	3	Somewhat agree	132	17.7%
	4	Neutral (neither disagree nor agree)	140	18.8%
	5	Somewhat disagree	104	13.9%
	6	Moderately disagree	87	11.7%
	7	Strongly disagree	100	13.4%

respondmissing

		Value	Count	Percent
Standard Attributes	Label	To what extent do you agree or disagree with the following statements? If I received a UF Alert about an on campus __________________________, I would know how to respond. - Missing Person		
N	Valid	743		
	Missing	3		
Central Tendency and Dispersion	Mean	3.75		
	Standard Deviation	1.732		
Labeled Values	1	Strongly agree	89	11.9%
	2	Moderately agree	107	14.3%
	3	Somewhat agree	127	17.0%
	4	Neutral (neither disagree nor agree)	190	25.5%
	5	Somewhat disagree	101	13.5%
	6	Moderately disagree	69	9.2%
	7	Strongly disagree	60	8.0%

respondsuicide

		Value	Count	Percent
Standard Attributes	Label	To what extent do you agree or disagree with the following statements? If I received a UF Alert about an on campus __________________________, I would know how to respond. - Suicide Threat or Attempt		
N	Valid	745		
	Missing	1		
Central Tendency and Dispersion	Mean	4.04		
	Standard Deviation	1.773		
Labeled Values	1	Strongly agree	77	10.3%
	2	Moderately agree	80	10.7%
	3	Somewhat agree	117	15.7%
	4	Neutral (neither disagree nor agree)	183	24.5%
	5	Somewhat disagree	121	16.2%
	6	Moderately disagree	83	11.1%
	7	Strongly disagree	84	11.3%

respondutility

		Value	Count	Percent
Standard Attributes	Label	To what extent do you agree or disagree with the following statements? If I received a UF Alert about an on campus __________________________, I would know how to respond. - Utility Failure		
N	Valid	742		
	Missing	4		
Central Tendency and Dispersion	Mean	4.07		
	Standard Deviation	1.756		
Labeled Values	1	Strongly agree	66	8.8%
	2	Moderately agree	87	11.7%
	3	Somewhat agree	124	16.6%
	4	Neutral (neither disagree nor agree)	170	22.8%
	5	Somewhat disagree	131	17.6%
	6	Moderately disagree	77	10.3%
	7	Strongly disagree	87	11.7%

respondbomb

		Value	Count	Percent
Standard Attributes	Label	To what extent do you agree or disagree with the following statements? If I received a UF Alert about an on campus __________________________, I would know how to respond. - Bomb Threat		
N	Valid	739		
	Missing	7		
Central Tendency and Dispersion	Mean	3.86		
	Standard Deviation	1.941		
Labeled Values	1	Strongly agree	118	15.8%
	2	Moderately agree	89	11.9%
	3	Somewhat agree	121	16.2%
	4	Neutral (neither disagree nor agree)	121	16.2%
	5	Somewhat disagree	118	15.8%
	6	Moderately disagree	82	11.0%
	7	Strongly disagree	90	12.1%

respondfire

		Value	Count	Percent
Standard Attributes	Label	To what extent do you agree or disagree with the following statements? If I received a UF Alert about an on campus __________________________, I would know how to respond. - Fire		
N	Valid	742		
	Missing	4		
Central Tendency and Dispersion	Mean	3.02		
	Standard Deviation	1.843		
Labeled Values	1	Strongly agree	197	26.4%
	2	Moderately agree	147	19.7%
	3	Somewhat agree	144	19.3%
	4	Neutral (neither disagree nor agree)	103	13.8%
	5	Somewhat disagree	56	7.5%
	6	Moderately disagree	38	5.1%
	7	Strongly disagree	57	7.6%

respondgunman

		Value	Count	Percent
Standard Attributes	Label	To what extent do you agree or disagree with the following statements? If I received a UF Alert about an on campus __________________________, I would know how to respond. - Gunman on Campus		
N	Valid	744		
	Missing	2		
Central Tendency and Dispersion	Mean	3.73		
	Standard Deviation	1.958		
Labeled Values	1	Strongly agree	128	17.2%
	2	Moderately agree	97	13.0%
	3	Somewhat agree	138	18.5%
	4	Neutral (neither disagree nor agree)	122	16.4%
	5	Somewhat disagree	96	12.9%
	6	Moderately disagree	68	9.1%
	7	Strongly disagree	95	12.7%

respondtrespass

		Value	Count	Percent
Standard Attributes	Label	To what extent do you agree or disagree with the following statements? If I received a UF Alert about an on campus _________________________, I would know how to respond. - Trespass Notice		
N	Valid	742		
	Missing	4		
Central Tendency and Dispersion	Mean	3.88		
	Standard Deviation	1.766		
Labeled Values	1	Strongly agree	87	11.7%
	2	Moderately agree	90	12.1%
	3	Somewhat agree	130	17.4%
	4	Neutral (neither disagree nor agree)	168	22.5%
	5	Somewhat disagree	128	17.2%
	6	Moderately disagree	66	8.8%
	7	Strongly disagree	73	9.8%

respondtornado

		Value	Count	Percent
Standard Attributes	Label	To what extent do you agree or disagree with the following statements? If I received a UF Alert about an on campus __________________________, I would know how to respond. - Tornado		
N	Valid	744		
	Missing	2		
Central Tendency and Dispersion	Mean	3.63		
	Standard Deviation	1.894		
Labeled Values	1	Strongly agree	122	16.4%
	2	Moderately agree	114	15.3%
	3	Somewhat agree	150	20.1%
	4	Neutral (neither disagree nor agree)	112	15.0%
	5	Somewhat disagree	94	12.6%
	6	Moderately disagree	80	10.7%
	7	Strongly disagree	72	9.7%

respondinclement

		Value	Count	Percent
Standard Attributes	Label	To what extent do you agree or disagree with the following statements? If I received a UF Alert about an on campus __________________________, I would know how to respond. - Inclement Weather Closing		
N	Valid	741		
	Missing	5		
Central Tendency and Dispersion	Mean	3.42		
	Standard Deviation	1.871		
Labeled Values	1	Strongly agree	139	18.6%
	2	Moderately agree	129	17.3%
	3	Somewhat agree	154	20.6%
	4	Neutral (neither disagree nor agree)	108	14.5%
	5	Somewhat disagree	89	11.9%
	6	Moderately disagree	54	7.2%
	7	Strongly disagree	68	9.1%

easeofuse1

		Value	Count	Percent
Standard Attributes	Label	To what extent do you agree or disagree with the following statements? - I think that UF Alerts are easy to use.		
N	Valid	744		
	Missing	2		
Central Tendency and Dispersion	Mean	2.13		
	Standard Deviation	1.217		
Labeled Values	1	Strongly agree	300	40.2%
	2	Moderately agree	206	27.6%
	3	Somewhat agree	121	16.2%
	4	Neutral (neither disagree nor agree)	89	11.9%
	5	Somewhat disagree	20	2.7%
	6	Moderately disagree	5	0.7%
	7	Strongly disagree	3	0.4%

easeofuse2

		Value	Count	Percent
Standard Attributes	Label	To what extent do you agree or disagree with the following statements? - I think that UF Alerts are clear and understandable.		
N	Valid	743		
	Missing	3		
Central Tendency and Dispersion	Mean	2.38		
	Standard Deviation	1.387		
Labeled Values	1	Strongly agree	251	33.6%
	2	Moderately agree	194	26.0%
	3	Somewhat agree	159	21.3%
	4	Neutral (neither disagree nor agree)	67	9.0%
	5	Somewhat disagree	52	7.0%
	6	Moderately disagree	12	1.6%
	7	Strongly disagree	8	1.1%

easeofuse3

		Value	Count	Percent
Standard Attributes	Label	To what extent do you agree or disagree with the following statements? - Learning how to use the UF Alert system was easy for me.		
N	Valid	743		
	Missing	3		
Central Tendency and Dispersion	Mean	2.52		
	Standard Deviation	1.482		
Labeled Values	1	Strongly agree	254	34.0%
	2	Moderately agree	168	22.5%
	3	Somewhat agree	106	14.2%
	4	Neutral (neither disagree nor agree)	144	19.3%
	5	Somewhat disagree	46	6.2%
	6	Moderately disagree	16	2.1%
	7	Strongly disagree	9	1.2%

easeofuse4

		Value	Count	Percent
Standard Attributes	Label	To what extent do you agree or disagree with the following statements? - The UF Alert system is easy to interact with.		
N	Valid	743		
	Missing	3		
Central Tendency and Dispersion	Mean	3.01		
	Standard Deviation	1.644		
Labeled Values	1	Strongly agree	193	25.9%
	2	Moderately agree	128	17.2%
	3	Somewhat agree	102	13.7%
	4	Neutral (neither disagree nor agree)	197	26.4%
	5	Somewhat disagree	69	9.2%
	6	Moderately disagree	33	4.4%
	7	Strongly disagree	21	2.8%

usefulness1

		Value	Count	Percent
Standard Attributes	Label	To what extent do you agree or disagree with the following statements? - As a student at the University of Florida (UF), I find that UF Alerts are useful.		
N	Valid	745		
	Missing	1		
Central Tendency and Dispersion	Mean	2.12		
	Standard Deviation	1.306		
Labeled Values	1	Strongly agree	318	42.6%
	2	Moderately agree	199	26.7%
	3	Somewhat agree	121	16.2%
	4	Neutral (neither disagree nor agree)	61	8.2%
	5	Somewhat disagree	29	3.9%
	6	Moderately disagree	10	1.3%
	7	Strongly disagree	7	0.9%

usefulness2

		Value	Count	Percent
Standard Attributes	Label	To what extent do you agree or disagree with the following statements? - The UF Alert system enables me to access the most RECENT updates about emergency situations occurring on or near the University of Florida campus.		
N	Valid	745		
	Missing	1		
Central Tendency and Dispersion	Mean	2.21		
	Standard Deviation	1.320		
Labeled Values	1	Strongly agree	302	40.5%
	2	Moderately agree	179	24.0%
	3	Somewhat agree	134	18.0%
	4	Neutral (neither disagree nor agree)	93	12.5%
	5	Somewhat disagree	18	2.4%
	6	Moderately disagree	13	1.7%
	7	Strongly disagree	6	0.8%

usefulness3

		Value	Count	Percent
Standard Attributes	Label	To what extent do you agree or disagree with the following statements? - The UF Alert system enables me to access the most ACCURATE updates about emergency situations occurring on or near the University of Florida campus.		
N	Valid	745		
	Missing	1		
Central Tendency and Dispersion	Mean	2.31		
	Standard Deviation	1.309		
Labeled Values	1	Strongly agree	267	35.8%
	2	Moderately agree	186	24.9%
	3	Somewhat agree	142	19.0%
	4	Neutral (neither disagree nor agree)	113	15.1%
	5	Somewhat disagree	24	3.2%
	6	Moderately disagree	6	0.8%
	7	Strongly disagree	7	0.9%

usefulness4

		Value	Count	Percent
Standard Attributes	Label	To what extent do you agree or disagree with the following statements? - If I could, I would unsubscribe from the UF Alert email system.		
N	Valid	743		
	Missing	3		
Central Tendency and Dispersion	Mean	4.89		
	Standard Deviation	1.996		
Labeled Values	1	Strongly agree	68	9.1%
	2	Moderately agree	47	6.3%
	3	Somewhat agree	65	8.7%
	4	Neutral (neither disagree nor agree)	124	16.6%
	5	Somewhat disagree	94	12.6%
	6	Moderately disagree	103	13.8%
	7	Strongly disagree	242	32.4%

usefulness5

		Value	Count	Percent
Standard Attributes	Label	To what extent do you agree or disagree with the following statements? - The University of Florida needs an emergency notification system to inform students, faculty, and staff about emergency incidents.		
N	Valid	744		
	Missing	2		
Central Tendency and Dispersion	Mean	2.11		
	Standard Deviation	1.406		
Labeled Values	1	Strongly agree	373	50.0%
	2	Moderately agree	131	17.6%
	3	Somewhat agree	93	12.5%
	4	Neutral (neither disagree nor agree)	112	15.0%
	5	Somewhat disagree	15	2.0%
	6	Moderately disagree	8	1.1%
	7	Strongly disagree	12	1.6%

attitudeUFhomepage

		Value	Count	Percent
Standard Attributes	Label	To what extent do you agree or disagree with the following statement? Sending a UF Alert through each of the following channels a good idea. - The University of Florida homepage		
N	Valid	742		
	Missing	4		
Central Tendency and Dispersion	Mean	2.65		
	Standard Deviation	1.780		
Labeled Values	1	Strongly agree	290	38.9%
	2	Moderately agree	121	16.2%
	3	Somewhat agree	121	16.2%
	4	Neutral (neither disagree nor agree)	78	10.5%
	5	Somewhat disagree	61	8.2%
	6	Moderately disagree	43	5.8%
	7	Strongly disagree	28	3.8%

attitudetext

		Value	Count	Percent
Standard Attributes	Label	To what extent do you agree or disagree with the following statement? Sending a UF Alert through each of the following channels a good idea. - Text messaging		
N	Valid	741		
	Missing	5		
Central Tendency and Dispersion	Mean	1.52		
	Standard Deviation	1.011		
Labeled Values	1	Strongly agree	527	70.6%
	2	Moderately agree	119	16.0%
	3	Somewhat agree	41	5.5%
	4	Neutral (neither disagree nor agree)	39	5.2%
	5	Somewhat disagree	8	1.1%
	6	Moderately disagree	5	0.7%
	7	Strongly disagree	2	0.3%

attitudeemail

		Value	Count	Percent
Standard Attributes	Label	To what extent do you agree or disagree with the following statement? Sending a UF Alert through each of the following channels a good idea. - Email		
N	Valid	739		
	Missing	7		
Central Tendency and Dispersion	Mean	1.84		
	Standard Deviation	1.234		
Labeled Values	1	Strongly agree	423	56.7%
	2	Moderately agree	145	19.4%
	3	Somewhat agree	87	11.7%
	4	Neutral (neither disagree nor agree)	55	7.4%
	5	Somewhat disagree	15	2.0%
	6	Moderately disagree	8	1.1%
	7	Strongly disagree	6	0.8%

attitudeclassphones

		Value	Count	Percent
Standard Attributes	Label	To what extent do you agree or disagree with the following statement? Sending a UF Alert through each of the following channels a good idea. - IP telephones and speakers (i.e. phones and speakers installed in classrooms)		
N	Valid	741		
	Missing	5		
Central Tendency and Dispersion	Mean	2.41		
	Standard Deviation	1.563		
Labeled Values	1	Strongly agree	297	39.8%
	2	Moderately agree	154	20.6%
	3	Somewhat agree	112	15.0%
	4	Neutral (neither disagree nor agree)	103	13.8%
	5	Somewhat disagree	41	5.5%
	6	Moderately disagree	12	1.6%
	7	Strongly disagree	22	2.9%

attitudefacebook

		Value	Count	Percent
Standard Attributes	Label	To what extent do you agree or disagree with the following statement? Sending a UF Alert through each of the following channels a good idea. - Facebook		
N	Valid	745		
	Missing	1		
Central Tendency and Dispersion	Mean	2.74		
	Standard Deviation	1.705		
Labeled Values	1	Strongly agree	247	33.1%
	2	Moderately agree	143	19.2%
	3	Somewhat agree	116	15.5%
	4	Neutral (neither disagree nor agree)	123	16.5%
	5	Somewhat disagree	63	8.4%
	6	Moderately disagree	20	2.7%
	7	Strongly disagree	33	4.4%

attitudetwitter

		Value	Count	Percent
Standard Attributes	Label	To what extent do you agree or disagree with the following statement? Sending a UF Alert through each of the following channels a good idea. - Twitter		
N	Valid	742		
	Missing	4		
Central Tendency and Dispersion	Mean	2.85		
	Standard Deviation	1.754		
Labeled Values	1	Strongly agree	233	31.2%
	2	Moderately agree	139	18.6%
	3	Somewhat agree	106	14.2%
	4	Neutral (neither disagree nor agree)	139	18.6%
	5	Somewhat disagree	61	8.2%
	6	Moderately disagree	26	3.5%
	7	Strongly disagree	38	5.1%

attituderssfeed

		Value	Count	Percent
Standard Attributes	Label	To what extent do you agree or disagree with the following statement? Sending a UF Alert through each of the following channels a good idea. - RSS feed		
N	Valid	739		
	Missing	7		
Central Tendency and Dispersion	Mean	3.05		
	Standard Deviation	1.698		
Labeled Values	1	Strongly agree	190	25.5%
	2	Moderately agree	121	16.2%
	3	Somewhat agree	112	15.0%
	4	Neutral (neither disagree nor agree)	197	26.4%
	5	Somewhat disagree	57	7.6%
	6	Moderately disagree	24	3.2%
	7	Strongly disagree	38	5.1%

attitudeUFrumorcontrol

		Value	Count	Percent
Standard Attributes	Label	To what extent do you agree or disagree with the following statement? Sending a UF Alert through each of the following channels a good idea. - University of Florida Rumor Control Hotline		
N	Valid	740		
	Missing	6		
Central Tendency and Dispersion	Mean	3.06		
	Standard Deviation	1.755		
Labeled Values	1	Strongly agree	202	27.1%
	2	Moderately agree	111	14.9%
	3	Somewhat agree	111	14.9%
	4	Neutral (neither disagree nor agree)	185	24.8%
	5	Somewhat disagree	64	8.6%
	6	Moderately disagree	22	2.9%
	7	Strongly disagree	45	6.0%

attitudereliable

		Value	Count	Percent
Standard Attributes	Label	Which of the following words reflect your perception of the UF Alert system? Select all that apply. - Selected Choice Reliable		
Valid Values	1	Reliable	396	53.1%
Missing Values	System		350	46.9%

attitudevaluable

		Value	Count	Percent
Standard Attributes	Label	Which of the following words reflect your perception of the UF Alert system? Select all that apply. - Selected Choice Valuable		
Valid Values	1	Valuable	379	50.8%
Missing Values	System		367	49.2%

attitudeinformative

		Value	Count	Percent
Standard Attributes	Label	Which of the following words reflect your perception of the UF Alert system? Select all that apply. - Selected Choice Informative		
Valid Values	1	Informative	611	81.9%
Missing Values	System		135	18.1%

attitudeclear

		Value	Count	Percent
Standard Attributes	Label	Which of the following words reflect your perception of the UF Alert system? Select all that apply. - Selected Choice Clear		
Valid Values	1	Clear	299	40.1%
Missing Values	System		447	59.9%

attitudehonest

		Value	Count	Percent
Standard Attributes	Label	Which of the following words reflect your perception of the UF Alert system? Select all that apply. - Selected Choice Honest		
Valid Values	1	Honest	238	31.9%
Missing Values	System		508	68.1%

attitudeuseful

		Value	Count	Percent
Standard Attributes	Label	Which of the following words reflect your perception of the UF Alert system? Select all that apply. - Selected Choice Useful		
Valid Values	1	Useful	407	54.6%
Missing Values	System		339	45.4%

attitudetimely

		Value	Count	Percent
Standard Attributes	Label	Which of the following words reflect your perception of the UF Alert system? Select all that apply. - Selected Choice Timely		
Valid Values	1	Timely	319	42.8%
Missing Values	System		427	57.2%

attitudeefficient

		Value	Count	Percent
Standard Attributes	Label	Which of the following words reflect your perception of the UF Alert system? Select all that apply. - Selected Choice Efficient		
Valid Values	1	Efficient	258	34.6%
Missing Values	System		488	65.4%

attitudestraightforward

		Value	Count	Percent
Standard Attributes	Label	Which of the following words reflect your perception of the UF Alert system? Select all that apply. - Selected Choice Straight-forward		
Valid Values	1	Straight-forward	402	53.9%
Missing Values	System		344	46.1%

attitudeimportant

		Value	Count	Percent
Standard Attributes	Label	Which of the following words reflect your perception of the UF Alert system? Select all that apply. - Selected Choice Important		
N	Valid	471		
	Missing	275		
Labeled Values	1	Important	471	63.1%

attitudedetailed

		Value	Count	Percent
Standard Attributes	Label	Which of the following words reflect your perception of the UF Alert system? Select all that apply. - Selected Choice Detailed		
N	Valid	133		
	Missing	613		
Labeled Values	1	Detailed	133	17.8%

attitudenoneoftheabovepositive

		Value	Count	Percent
Standard Attributes	Label	Which of the following words reflect your perception of the UF Alert system? Select all that apply. - Selected Choice None of the above		
N	Valid	12		
	Missing	734		
Labeled Values	1	None of the above	12	1.6%

attitudeotherpositive

		Value	Count	Percent
Standard Attributes	Label	Which of the following words reflect your perception of the UF Alert system? Select all that apply. - Selected Choice Other		
N	Valid	7		
	Missing	739		
Labeled Values	1	Other	7	0.9%

attitudeotherpositivetext

		Value	Count	Percent
Standard Attributes	Label	Which of the following words reflect your perception of the UF Alert system? Select all that apply. - Other - Text		
Valid Values			741	99.3%
	Annoying		1	0.1%
	Biased		1	0.1%
	I don't attend school on campus		1	0.1%
	I live off campus so I would say it's unnecessary.		1	0.1%
	Not Applicable to me		1	0.1%

attitudeworthless

		Value	Count	Percent
Standard Attributes	Label	Which of the following words reflect your perception of the UF Alert system? Select all that apply. - Selected Choice Worthless		
N	Valid	14		
	Missing	732		
Labeled Values	1	Worthless	14	1.9%

attitudeuninformative

		Value	Count	Percent
Standard Attributes	Label	Which of the following words reflect your perception of the UF Alert system? Select all that apply. - Selected Choice Uninformative		
Valid Values	1	Uninformative	16	2.1%
Missing Values	System		730	97.9%

attitudeconfusing

		Value	Count	Percent
Standard Attributes	Label	Which of the following words reflect your perception of the UF Alert system? Select all that apply. - Selected Choice Confusing		
Valid Values	1	Confusing	117	15.7%
Missing Values	System		629	84.3%

attitudedishonest

		Value	Count	Percent
Standard Attributes	Label	Which of the following words reflect your perception of the UF Alert system? Select all that apply. - Selected Choice Dishonest		
Valid Values	1	Dishonest	4	0.5%
Missing Values	System		742	99.5%

attitudeunpleasant

		Value	Count	Percent
Standard Attributes	Label	Which of the following words reflect your perception of the UF Alert system? Select all that apply. - Selected Choice Unpleasant		
Valid Values	1	Unpleasant	35	4.7%
Missing Values	System		711	95.3%

attitudeannoying

		Value	Count	Percent
Standard Attributes	Label	Which of the following words reflect your perception of the UF Alert system? Select all that apply. - Selected Choice Annoying		
Valid Values	1	Annoying	157	21.0%
Missing Values	System		589	79.0%

attitudeunreliable

		Value	Count	Percent
Standard Attributes	Label	Which of the following words reflect your perception of the UF Alert system? Select all that apply. - Selected Choice Unreliable		
Valid Values	1	Unreliable	27	3.6%
Missing Values	System		719	96.4%

attituderedundant

		Value	Count	Percent
Standard Attributes	Label	Which of the following words reflect your perception of the UF Alert system? Select all that apply. - Selected Choice Redundant		
Valid Values	1	Redundant	151	20.2%
Missing Values	System		595	79.8%

attitudeinefficient

		Value	Count	Percent
Standard Attributes	Label	Which of the following words reflect your perception of the UF Alert system? Select all that apply. - Selected Choice Inefficient		
Valid Values	1	Inefficient	62	8.3%
Missing Values	System		684	91.7%

attitudescary

		Value	Count	Percent
Standard Attributes	Label	Which of the following words reflect your perception of the UF Alert system? Select all that apply. - Selected Choice Scary		
Valid Values	1	Scary	168	22.5%
Missing Values	System		578	77.5%

attitudevague

		Value	Count	Percent
Standard Attributes	Label	Which of the following words reflect your perception of the UF Alert system? Select all that apply. - Selected Choice Vague		
Valid Values	1	Vague	202	27.1%
Missing Values	System		544	72.9%

attitudenoneoftheabove

		Value	Count	Percent
Standard Attributes	Label	Which of the following words reflect your perception of the UF Alert system? Select all that apply. - Selected Choice None of the above		
Valid Values	1	None of the above	179	24.0%
Missing Values	System		567	76.0%

attitudeother

		Value	Count	Percent
Standard Attributes	Label	Which of the following words reflect your perception of the UF Alert system? Select all that apply. - Selected Choice Other		
Valid Values	1	Other	15	2.0%
Missing Values	System		731	98.0%

attitudeothertext

		Value	Count	Percent
Standard Attributes	Label	Which of the following words reflect your perception of the UF Alert system? Select all that apply. - Other - Text		
Valid Values			734	98.4%
	conflicting, too brief		1	0.1%
	I live in Orlando and the alerts have nothing to do with me.		1	0.1%
	leans towards Black fugitives/criminals		1	0.1%
	never know if/when the situation is resolved and i feel unsafe		1	0.1%
	Not Applicable to me		1	0.1%
	repetitive		1	0.1%
	sometimes slightly annoying but I recognize that they're important, sometimes the shorthand is confusing but I usually get the gist		1	0.1%
	they never tell you the outcome if it's negative- like they wont text you saying the suspect has not been found		1	0.1%
	un clear		1	0.1%
	untimely		1	0.1%
	Untimely		1	0.1%
	Usually end with "suspect still at large"		1	0.1%

intendread1

		Value	Count	Percent
Standard Attributes	Label	To what extent do you agree or disagree with the following statements? - I intend to read all future UF Alert messages I receive.		
N	Valid	744		
	Missing	2		
Central Tendency and Dispersion	Mean	1.97		
	Standard Deviation	1.286		
Labeled Values	1	Strongly agree	370	49.6%
	2	Moderately agree	167	22.4%
	3	Somewhat agree	131	17.6%
	4	Neutral (neither disagree nor agree)	39	5.2%
	5	Somewhat disagree	17	2.3%
	6	Moderately disagree	9	1.2%
	7	Strongly disagree	11	1.5%

intendfollow2

		Value	Count	Percent
Standard Attributes	Label	To what extent do you agree or disagree with the following statements? - I intend to "follow" UF Alerts on Twitter.		
N	Valid	742		
	Missing	4		
Central Tendency and Dispersion	Mean	4.70		
	Standard Deviation	1.940		
Labeled Values	1	Strongly agree	62	8.3%
	2	Moderately agree	57	7.6%
	3	Somewhat agree	58	7.8%
	4	Neutral (neither disagree nor agree)	188	25.2%
	5	Somewhat disagree	94	12.6%
	6	Moderately disagree	67	9.0%
	7	Strongly disagree	216	29.0%

intendlike3

		Value	Count	Percent
Standard Attributes	Label	To what extent do you agree or disagree with the following statements? - I intend to "like" UF Alerts on Facebook.		
N	Valid	744		
	Missing	2		
Central Tendency and Dispersion	Mean	4.37		
	Standard Deviation	1.982		
Labeled Values	1	Strongly agree	85	11.4%
	2	Moderately agree	67	9.0%
	3	Somewhat agree	84	11.3%
	4	Neutral (neither disagree nor agree)	165	22.1%
	5	Somewhat disagree	108	14.5%
	6	Moderately disagree	64	8.6%
	7	Strongly disagree	171	22.9%

intendunsubscribe4

		Value	Count	Percent
Standard Attributes	Label	To what extent do you agree or disagree with the following statements? - I intend to unsubscribe from UF Alert text messages.		
N	Valid	741		
	Missing	5		
Central Tendency and Dispersion	Mean	5.62		
	Standard Deviation	1.689		
Labeled Values	1	Strongly agree	32	4.3%
	2	Moderately agree	23	3.1%
	3	Somewhat agree	22	2.9%
	4	Neutral (neither disagree nor agree)	102	13.7%
	5	Somewhat disagree	100	13.4%
	6	Moderately disagree	119	16.0%
	7	Strongly disagree	343	46.0%

intendignore5

		Value	Count	Percent
Standard Attributes	Label	To what extent do you agree or disagree with the following statements? - I intend to ignore all future UF Alert messages.		
N	Valid	742		
	Missing	4		
Central Tendency and Dispersion	Mean	5.85		
	Standard Deviation	1.528		
Labeled Values	1	Strongly agree	18	2.4%
	2	Moderately agree	19	2.5%
	3	Somewhat agree	16	2.1%
	4	Neutral (neither disagree nor agree)	90	12.1%
	5	Somewhat disagree	105	14.1%
	6	Moderately disagree	109	14.6%
	7	Strongly disagree	385	51.6%

Demographics and Characteristics

sex

		Value	Count	Percent
Standard Attributes	Label	What is your sex?		
Valid Values	1	Male	199	26.7%
	2	Female	544	72.9%
	3	Intersex/Transexual/Genderqueer	2	0.3%
Missing Values	System		1	0.1%

military

		Value	Count	Percent
Standard Attributes	Label	Have you ever served on active duty in the U.S. Armed Forces, military Reserves, or National Guard? Active Duty does not include training for the Reserves or National Guard, but DOES include activation, for example, for the Persian Gulf War.		
Valid Values	1	Yes, now on active duty	4	0.5%
	2	Yes, on active duty during the last 12 months, but not now	3	0.4%
	3	Yes, on active duty in the past, but not during the last 12 months	7	0.9%
	4	No, training for Reserves or National Guard only	9	1.2%
	5	No, never served in the military	722	96.8%
Missing Values	System		1	0.1%

greeklife

		Value	Count	Percent
Standard Attributes	Label	Are you a member of a social fraternity or sorority?		
Valid Values	1	Yes	197	26.4%
	2	No	539	72.3%
	3	I am in the process of pledging/rushing/recruitment this semester	10	1.3%

ethnicity

		Value	Count	Percent
Standard Attributes	Label	Ethnicity		
Valid Values	1	White	518	69.4%
	2	Black or African American	97	13.0%
	3	American Indian or Alaska Native	1	0.1%
	4	Asian Indian	24	3.2%
	5	Chinese	13	1.7%
	6	Filipino	8	1.1%
	7	Japanese	2	0.3%
	8	Korean	6	0.8%
	9	Vietnamese	10	1.3%
	10	Other Asian	5	0.7%
	14	Other Pacific Islander	7	0.9%
	15	Multiple	39	5.2%
Missing Values	System		16	2.1%

hispanic

		Value	Count	Percent
Standard Attributes	Label	Hispanic/Latino/a/Spanish origin		
Valid Values	1	Not Hispanic/Latino/Spanish origin	598	80.2%
	2	Mexican, Mexican American, Chicano/a	11	1.5%
	3	Puerto Rican	16	2.1%
	4	Cuban	44	5.9%
	5	Another Hispanic/Latino/Spanish origin	59	7.9%
	6	Multiple	14	1.9%
Missing Values	System		4	0.5%

sexualorientation

		Value	Count	Percent
Standard Attributes	Label	How would you classify your sexual orientation?		
Valid Values	1	Asexual	5	0.7%
	2	Bisexual/Bi	10	1.3%
	3	Heterosexual/Straight	700	93.8%
	4	Homosexual/Gay/Lesbian/Queer	17	2.3%
	5	Unsure	5	0.7%
	6	Decline to answer	9	1.2%

relationshipstatus

		Value	Count	Percent
Standard Attributes	Label	What is your current relationship status? - Selected Choice		
Valid Values	1	Married	23	3.1%
	2	In a committed relationship (with a steady partner)	236	31.6%
	3	Single (not dating)	362	48.5%
	4	Dating	110	14.7%
	5	Divorced	5	0.7%
	6	Widowed	2	0.3%
	7	Separated	3	0.4%
	8	Other	5	0.7%

relationshiptext

		Value	Count	Percent
Standard Attributes	Label	What is your current relationship status? - Other - Text		
Valid Values			742	99.5%
	Engaged		2	0.3%
	exclusive relationship but not officially dating		1	0.1%
	I mingle from time to time		1	0.1%

classification

		Value	Count	Percent
Standard Attributes	Label	What is your classification at the University of Florida?		
Valid Values	1	Freshman	94	12.6%
	2	Sophomore	221	29.6%
	3	Junior	231	31.0%
	4	Senior	200	26.8%
	5	Graduate Student	0	0.0%
	6	Professional Student	0	0.0%
	7	Non-degree seeking student	0	0.0%
	8	I am not a student at the University of Florida	0	0.0%

In which college is your current major?

		Frequency	Percent	Valid Percent	Cumulative Percent
Valid	College of Agricultural and Life Sciences	82	11.0	11.0	11.0
	College of Business Administration	21	2.8	2.8	13.8
	College of Design, Construction, and Planning	4	.5	.5	14.4
	College of Education	3	.4	.4	14.8
	College of Engineering	12	1.6	1.6	16.4
	College of Fine Arts	5	.7	.7	17.0
	College of Health and Human Performance	185	24.8	24.8	41.9
	College of Journalism and Communications	25	3.4	3.4	45.2
	College of Liberal Arts and Sciences	253	33.9	34.0	79.2
	College of Medicine	3	.4	.4	79.6
	College of Nursing	58	7.8	7.8	87.4
	College of Pharmacy	4	.5	.5	87.9
	College of Public Health and Health Professions	90	12.1	12.1	100.0
	Total	745	99.9	100.0	
Missing	System	1	.1		
Total		746	100.0		

currentresidence

		Value	Count	Percent
Standard Attributes	Label	Where do you currently live?		
Valid Values	1	On campus dormitory	135	18.1%
	2	Off-campus dormitory	16	2.1%
	3	Apartment	420	56.3%
	4	House	156	20.9%
	5	Other	18	2.4%
Missing Values	System		1	0.1%

livewithparents

		Value	Count	Percent
Standard Attributes	Label	Do you live with your parent(s) or guardian(s)?		
Valid Values	1	Yes	70	9.4%
	2	No	675	90.5%
Missing Values	System		1	0.1%

racewhite

		Value	Count	Percent
Standard Attributes	Label	What is your race? (One or more categories may be selected) White		
N	Valid	746		
	Missing	0		
Labeled Values	1	White	552	74.0%

raceblackafricanamerican

		Value	Count	Percent
Standard Attributes	Label	What is your race? (One or more categories may be selected) Black or African American		
N	Valid	746		
	Missing	0		
Labeled Values	1	Black or African American	110	14.7%

raceamericanindianalaskanative

		Value	Count	Percent
Standard Attributes	Label	What is your race? (One or more categories may be selected) American Indian or Alaska Native		
N	Valid	746		
	Missing	0		
Labeled Values	1	American Indian or Alaska Native	13	1.7%

raceasianindian

		Value	Count	Percent
Standard Attributes	Label	What is your race? (One or more categories may be selected) Asian Indian		
N	Valid	746		
	Missing	0		
Labeled Values	1	Asian Indian	25	3.4%

racechinese

		Value	Count	Percent
Standard Attributes	Label	What is your race? (One or more categories may be selected) Chinese		
N	Valid	746		
	Missing	0		
Labeled Values	1	Chinese	16	2.1%

racefilipino

		Value	Count	Percent
Standard Attributes	Label	What is your race? (One or more categories may be selected) Filipino		
N	Valid	746		
	Missing	0		
Labeled Values	1	Filipino	15	2.0%

racejapanese

		Value	Count	Percent
Standard Attributes	Label	What is your race? (One or more categories may be selected) Japanese		
N	Valid	746		
	Missing	0		
Labeled Values	1	Japanese	6	0.8%

racekorean

		Value	Count	Percent
Standard Attributes	Label	What is your race? (One or more categories may be selected) Korean		
N	Valid	746		
	Missing	0		
Labeled Values	1	Korean	12	1.6%

racevietnamese

		Value	Count	Percent
Standard Attributes	Label	What is your race? (One or more categories may be selected) Vietnamese		
N	Valid	746		
	Missing	0		
Labeled Values	1	Vietnamese	12	1.6%

raceotherasian

		Value	Count	Percent
Standard Attributes	Label	What is your race? (One or more categories may be selected) Other Asian		
N	Valid	746		
	Missing	0		
Labeled Values	1	Other Asian	9	1.2%

racenativehawaiian

		Value	Count	Percent
Standard Attributes	Label	What is your race? (One or more categories may be selected) Native Hawaiian		
N	Valid	746		
	Missing	0		
Labeled Values	1	Native Hawaiian	2	0.3%

raceguamanianchamorro

		Value	Count	Percent
Standard Attributes	Label	What is your race? (One or more categories may be selected) Guamanian or Chamorro		
N	Valid	746		
	Missing	0		
Labeled Values	1	Guamanian or Chamorro	1	0.1%

racesamoan

		Value	Count	Percent
Standard Attributes	Label	What is your race? (One or more categories may be selected) Samoan		
N	Valid	746		
	Missing	0		
Labeled Values	1	Samoan	1	0.1%

raceotherpacificislander

		Value	Count	Percent
Standard Attributes	Label	What is your race? (One or more categories may be selected) Other Pacific Islander		
N	Valid	746		
	Missing	0		
Labeled Values	1	Other Pacific Islander	9	1.2%

hispanicno

		Value	Count	Percent
Standard Attributes	Label	Are you Hispanic, Latino/a, or Spanish Origin? (One or more categories may be selected) No, not of Hispanic, Latino/a, or Spanish origin		
N	Valid	746		
	Missing	0		
Labeled Values	1	No, not of Hispanic, Latino/a, or Spanish origin	599	80.3%

hispanicyesmexican

		Value	Count	Percent
Standard Attributes	Label	Are you Hispanic, Latino/a, or Spanish Origin? (One or more categories may be selected) Yes, Mexican, Mexican American, Chicano/a		
N	Valid	746		
	Missing	0		
Labeled Values	1	Yes, Mexican, Mexican American, Chicano/a	13	1.7%

hispanicyespuertorican

		Value	Count	Percent
Standard Attributes	Label	Are you Hispanic, Latino/a, or Spanish Origin? (One or more categories may be selected) Yes, Puerto Rican		
N	Valid	746		
	Missing	0		
Labeled Values	1	Yes, Puerto Rican	23	3.1%

hispanicyescuban

		Value	Count	Percent
Standard Attributes	Label	Are you Hispanic, Latino/a, or Spanish Origin? (One or more categories may be selected) Yes, Cuban		
N	Valid	746		
	Missing	0		
Labeled Values	1	Yes, Cuban	53	7.1%

hispanicyesother

		Value	Count	Percent
Standard Attributes	Label	Are you Hispanic, Latino/a, or Spanish Origin? (One or more categories may be selected) Yes, Another Hispanic, Latino/a, or Spanish origin		
N	Valid	746		
	Missing	0		
Labeled Values	1	Yes, Another Hispanic, Latino/a, or Spanish origin	69	9.2%

ownsmartphone

		Value	Count	Percent
Standard Attributes	Label	Do you currently own any of the following? - Cell phone with Internet capability (smart phone)		
N	Valid	744		
	Missing	2		
Labeled Values	1	Yes	687	92.1%
	2	No	57	7.6%

owncellphonewithoutinternet

		Value	Count	Percent
Standard Attributes	Label	Do you currently own any of the following? - Cell phone without Internet capability		
N	Valid	733		
	Missing	13		
Labeled Values	1	Yes	146	19.6%
	2	No	587	78.7%

owndesktop

		Value	Count	Percent
Standard Attributes	Label	Do you currently own any of the following? - Desktop computer		
N	Valid	735		
	Missing	11		
Labeled Values	1	Yes	196	26.3%
	2	No	539	72.3%

ownebookreader

		Value	Count	Percent
Standard Attributes	Label	Do you currently own any of the following? - Electronic book reader (e.g. Nook, Kindle)		
N	Valid	734		
	Missing	12		
Labeled Values	1	Yes	177	23.7%
	2	No	557	74.7%

owngameconsole

		Value	Count	Percent
Standard Attributes	Label	Do you currently own any of the following? - Game console (e.g. X-Box, PlayStation, Nintendo Wii)		
N	Valid	736		
	Missing	10		
Labeled Values	1	Yes	306	41.0%
	2	No	430	57.6%

ownlaptop

		Value	Count	Percent
Standard Attributes	Label	Do you currently own any of the following? - Laptop computer		
N	Valid	743		
	Missing	3		
Labeled Values	1	Yes	724	97.1%
	2	No	19	2.5%

ownmp3

		Value	Count	Percent
Standard Attributes	Label	Do you currently own any of the following? - mp3 player (e.g. iPod)		
N	Valid	738		
	Missing	8		
Labeled Values	1	Yes	590	79.1%
	2	No	148	19.8%

ownnetbook

		Value	Count	Percent
Standard Attributes	Label	Do you currently own any of the following? - Netbook computer		
N	Valid	732		
	Missing	14		
Labeled Values	1	Yes	84	11.3%
	2	No	648	86.9%

ownportablegaming

		Value	Count	Percent
Standard Attributes	Label	Do you currently own any of the following? - Portable gaming device (e.g. Sony PSP, Nintendo 3DS)		
N	Valid	734		
	Missing	12		
Labeled Values	1	Yes	106	14.2%
	2	No	628	84.2%

owntablet

		Value	Count	Percent
Standard Attributes	Label	Do you currently own any of the following? - Tablet computer (e.g. iPad)		
N	Valid	733		
	Missing	13		
Labeled Values	1	Yes	180	24.1%
	2	No	553	74.1%

accesshighspeedinternet

		Value	Count	Percent
Standard Attributes	Label	Do you have access to a high-speed Internet connection at your current residence?		
N	Valid	745		
	Missing	1		
Labeled Values	1	Yes	728	97.6%
	2	No	9	1.2%
	3	I'm not sure	8	1.1%

accessunlimitedtextplan

		Value	Count	Percent
Standard Attributes	Label	Do you have an unlimited text message/SMS plan?		
N	Valid	745		
	Missing	1		
Labeled Values	1	Yes	706	94.6%
	2	No	29	3.9%
	3	I don't know	10	1.3%

textssentperday

		Value	Count	Percent
Standard Attributes	Label	Approximately how many text/SMS messages do you send per day?		
N	Valid	744		
	Missing	2		
Labeled Values	1	0-10	65	8.7%
	2	11-20	144	19.3%
	3	21-50	245	32.8%
	4	More than 50	257	34.5%
	5	I don't know	33	4.4%

textsreceivedperday

		Value	Count	Percent
Standard Attributes	Label	How many text/SMS messages do you receive per day?		
N	Valid	743		
	Missing	3		
Labeled Values	1	0-10	79	10.6%
	2	11-20	130	17.4%
	3	21-50	232	31.1%
	4	More than 50	266	35.7%
	5	I don't know	36	4.8%
